# Supplementary material for: Economic suitability of direct seeded rice across different geographies in India
Source: PLoS One. 2025 Apr 18;20(4):e0321472. doi: 10.1371/journal.pone.0321472 (PMC12007715; doi:10.1371/journal.pone.0321472)
Supplement: S1 Table — Effect of adopting DSR practices on income, production, and expenses associated with paddy cultivation in Uttar Pradesh. (DOCX) [file pone.0321472.s001.docx]

**Table S1.** Effect of adopting DSR practices on income, production, and expenses associated with paddy cultivation in Uttar Pradesh

|  | **NNM** | | | | | **KBM** | | | | | **RM** | | | | |
| --- | --- | --- | --- | --- | --- | --- | --- | --- | --- | --- | --- | --- | --- | --- | --- |
|  | **DSR adopters** | **DSR non-adopters** | **ATT** | **SE** | **Critical level of hidden bias** | **DSR adopters** | **DSR non-adopters** | **ATT** | **SE** | **Critical level of hidden bias** | **DSR adopters** | **DSR non-adopters** | **ATT** | **SE** | **Critical level of hidden bias** |
| Land preparation cost (Rs/acre) | 587 | 3446 | -2859 | 47.14 | 3.25-3.30 | 563 | 3515 | -2952 | 49.15 | 2.45-2.50 | 504 | 3419 | -2915 | 47.23 | 2.75-2.80 |
| Seed and seed treatment cost (Rs/acre) | 895 | 1145 | -250 | 65.24 | 2.55-2.60 | 911 | 1207 | -296 | 72.65 | 2.75-2.80 | 883 | 1176 | -293 | 75.14 | 2.45-2.50 |
| Crop establishment cost (Rs/acre) | 1138 | 2791 | -1653 | 214.65 | 3.75-3.80 | 1189 | 2675 | -1486 | 230.18 | 1.95-2.00 | 1143 | 2745 | -1602 | 222.18 | 1.85-1.90 |
| Total fertilizer cost (Rs/acre) | 2253 | 2578 | -325 | 81.54 | 2.75-2.80 | 2145 | 2683 | -538 | 88.75 | 3.25-3.30 | 2165 | 2702 | -537 | 80.19 | 2.35-2.40 |
| Irrigation cost (Rs/acre) | 1728 | 2308 | -580 | 76.28 | 2.50-2.55 | 1645 | 2289 | -644 | 72.19 | 2.45-2.50 | 1702 | 2367 | -665 | 74.18 | 2.15-2.20 |
| Weed control cost (Rs/acre) | 1626 | 1418 | 208 | 39.12 | 2.65-2.70 | 1593 | 1499 | 94 | 36.87 | 2.50-2.55 | 1675 | 1495 | 180 | 34.76 | 3.00-3.05 |
| Pest control cost (Rs/acre) | 1655 | 1280 | 375 | 56.91 | 1.95-2.00 | 1677 | 1275 | 402 | 61.13 | 2.65-2.70 | 1723 | 1304 | 419 | 65.78 | 2.15-2.20 |
| Harvesting cost (Rs/acre) | 2510 | 2567 | -57 | 54.23 | 2.25-2.30 | 2417 | 2489 | -72 | 59.17 | 2.00-2.05 | 2456 | 2503 | -47 | 54.87 | 2.00-2.05 |
| Post-harvest cost (Rs/acre) | 6598 | 6732 | -134 | 76.46 | 2.55-2.60 | 6444 | 6573 | -129 | 81.83 | 2.55-2.60 | 6489 | 6538 | -49 | 78.1 | 2.85-2.90 |
| Total labor (days/acre) | 27.54 | 35.08 | -7.54 | 1.13 | 2.45-2.50 | 28.01 | 36.18 | -8.17 | 1.11 | 2.65-2.70 | 27.77 | 36.13 | -8.36 | 0.89 | 2.65-2.70 |
| Family labor (days/acre) | 14.17 | 17.13 | -2.96 | 0.45 | 2.15-2.20 | 13.16 | 16.23 | -3.07 | 0.34 | 2.15-2.20 | 13.83 | 17.34 | -3.51 | 0.23 | 2.05-2.10 |
| Total cost (Rs/acre) | 18119 | 24532 | -6413 | 654 | 2.00-2.05 | 18456 | 25754 | -7298 | 707 | 2.50-2.55 | 19144 | 26034 | -6890 | 678 | 3.25-3.30 |
| Rice yield (kg/acre) | 1763 | 1709 | 54 | 19.18 | 2.55-2.60 | 1754 | 1689 | 65 | 61.8 | 2.55-2.60 | 1761 | 1706 | 55 | 63.87 | 2.10-2.15 |
| Income from rice (Rs/acre) | 19843 | 13888 | 5955 | 543 | 2.45-2.50 | 19877 | 13666 | 6211 | 592 | 2.75-2.80 | 19998 | 13451 | 6547 | 600 | 2.75-2.80 |
