# Supplementary material for: Economic suitability of direct seeded rice across different geographies in India
Source: PLoS One. 2025 Apr 18;20(4):e0321472. doi: 10.1371/journal.pone.0321472 (PMC12007715; doi:10.1371/journal.pone.0321472)
Supplement: S2 Table — Effect of adopting DSR practices on income, production, and expenses associated with paddy cultivation in Madhya Pradesh. (DOCX) [file pone.0321472.s002.docx]

**Table S2.** Effect of adopting DSR practices on income, production, and expenses associated with paddy cultivation in Madhya Pradesh

|  | **NNM** | | | | | **KBM** | | | | | **RM** | | | | |
| --- | --- | --- | --- | --- | --- | --- | --- | --- | --- | --- | --- | --- | --- | --- | --- |
|  | **DSR adopters** | **DSR non-adopters** | **ATT** | **SE** | **Critical level of hidden bias** | **DSR adopters** | **DSR non-adopters** | **ATT** | **SE** | **Critical level of hidden bias** | **DSR adopters** | **DSR non-adopters** | **ATT** | **SE** | **Critical level of hidden bias** |
| Land preparation cost (Rs/acre) | 866 | 2600 | -1734 | 48.35 | 2.45-2.50 | 808 | 2635 | -1827 | 47.89 | 2.75-2.80 | 833 | 2580 | -1747 | 51.13 | 2.25-2.30 |
| Seed and seed treatment cost (Rs/acre) | 1054 | 1555 | -501 | 76.16 | 2.25-2.30 | 956 | 1445 | -489 | 74.31 | 2.35-2.40 | 989 | 1634 | -645 | 77.19 | 2.45-2.50 |
| Crop establishment cost (Rs/acre) | 1528 | 3356 | -1828 | 244.18 | 2.65-2.70 | 1467 | 3288 | -1821 | 234.18 | 2.15-2.20 | 1565 | 3032 | -1467 | 256.12 | 2.50-2.55 |
| Total fertilizer cost (Rs/acre) | 2854 | 3567 | -713 | 113.23 | 1.95-2.00 | 2799 | 3603 | -804 | 110.91 | 2.25-2.30 | 2751 | 3550 | -799 | 108.23 | 2.45-2.45 |
| Irrigation cost (Rs/acre) | 905 | 1618 | -713 | 145.6 | 3.75-3.80 | 877 | 1653 | -776 | 134.72 | 2.75-2.80 | 881 | 1578 | -697 | 145.31 | 2.15-2.20 |
| Weed control cost (Rs/acre) | 1518 | 1234 | 284 | 65.13 | 3.25-3.30 | 1543 | 1188 | 355 | 67.89 | 2.55-2.60 | 1481 | 1198 | 283 | 66.34 | 2.00-2.05 |
| Pest control cost (Rs/acre) | 1220 | 1186 | 34 | 18.33 | 1.95-2.00 | 1243 | 1187 | 56 | 17.63 | 2.45-2.50 | 1178 | 1156 | 22 | 16.45 | 2.25-2.30 |
| Harvesting cost (Rs/acre) | 1805 | 1856 | -51 | 24.54 | 3.75-3.80 | 1820 | 1836 | -16 | 22.76 | 2.10-2.15 | 1789 | 1835 | -46 | 23.23 | 2.10-2.15 |
| Post-harvest cost (Rs/acre) | 6002 | 6344 | -342 | 176.32 | 3.75-3.80 | 5983 | 6431 | -448 | 156.81 | 2.05-2.10 | 5989 | 6234 | -245 | 164.98 | 2.65-2.70 |
| Total labor (days/acre) | 28.94 | 35.44 | -6.5 | 1.15 | 2.15-2.20 | 28.56 | 36.01 | -7.45 | 1.09 | 2.35-2.40 | 28.91 | 35.73 | -6.82 | 1.05 | 2.35-2.40 |
| Family labor (days/acre) | 13.11 | 16.33 | -3.22 | 0.56 | 2.35-2.40 | 13.18 | 16.23 | -3.05 | 0.48 | 2.15-2.20 | 13.44 | 16.63 | -3.19 | 0.41 | 2.15-2.20 |
| Total cost (Rs/acre) | 17681 | 23101 | -5420 | 455.78 | 2.10-2.15 | 17554 | 23279 | -5725 | 441.23 | 2.25-2.30 | 18021 | 23534 | -5513 | 445.32 | 2.25-2.30 |
| Rice yield (kg/acre) | 1804 | 1989 | -185 | 83.13 | 2.45-2.50 | 1794 | 1976 | -182 | 78.91 | 2.05-2.10 | 1764 | 1933 | -169 | 72.13 | 2.30-2.35 |
| Income from rice (Rs/acre) | 22434 | 16512 | 5922 | 333.24 | 2.35-2.40 | 22145 | 16476 | 5669 | 401.34 | 2.15-2.20 | 21989 | 16501 | 5488 | 431.56 | 2.25-2.30 |
