# Supplementary material for: Economic suitability of direct seeded rice across different geographies in India
Source: PLoS One. 2025 Apr 18;20(4):e0321472. doi: 10.1371/journal.pone.0321472 (PMC12007715; doi:10.1371/journal.pone.0321472)
Supplement: S3 Table — Effect of adopting DSR practices on income, production, and expenses associated with paddy cultivation in Andhra Pradesh. (DOCX) [file pone.0321472.s003.docx]

**Table S3**. Effect of adopting DSR practices on income, production, and expenses associated with paddy cultivation in Andhra Pradesh

|  | **NNM** | | | | | **KBM** | | | | | **RM** | | | | |
| --- | --- | --- | --- | --- | --- | --- | --- | --- | --- | --- | --- | --- | --- | --- | --- |
|  | **DSR adopters** | **DSR non-adopters** | **ATT** | **SE** | **Critical level of hidden bias** | **DSR adopters** | **DSR non-adopters** | **ATT** | **SE** | **Critical level of hidden bias** | **DSR adopters** | **DSR non-adopters** | **ATT** | **SE** | **Critical level of hidden bias** |
| Land preparation cost (Rs/acre) | 758 | 2345 | -1587 | 64.23 | 2.15-2.20 | 818 | 2327 | -1509 | 58.14 | 2.25-2.30 | 789 | 2453 | -1664 | 59.32 | 2.45-2.50 |
| Seed and seed treatment cost (Rs/acre) | 877 | 1381 | -504 | 56.19 | 2.15-2.20 | 878 | 1425 | -547 | 58.24 | 2.35-2.40 | 902 | 1452 | -550 | 67.37 | 2.00-2.15 |
| Crop establishment cost (Rs/acre) | 1480 | 3576 | -2096 | 261.76 | 2.55-2.60 | 1522 | 3661 | -2139 | 281.83 | 2.00-2.05 | 1505 | 3632 | -2127 | 255.12 | 2.50-2.55 |
| Total fertilizer cost (Rs/acre) | 2943 | 3234 | -291 | 60.23 | 1.85-1.90 | 2907 | 3267 | -360 | 45.13 | 2.15-2.20 | 2952 | 3307 | -355 | 48.23 | 2.35-2.40 |
| Irrigation cost (Rs/acre) | 1655 | 2755 | -1100 | 211.43 | 2.75-2.80 | 1706 | 2798 | -1092 | 202.45 | 2.35-2.40 | 1695 | 2779 | -1084 | 214.6 | 2.20-2.25 |
| Weed control cost (Rs/acre) | 1777 | 1540 | 237 | 41.33 | 3.25-3.30 | 1721 | 1523 | 198 | 42.34 | 2.65-2.70 | 1785 | 1498 | 287 | 49.34 | 2.25-2.30 |
| Pest control cost (Rs/acre) | 2243 | 1931 | 312 | 69.17 | 2.00-2.05 | 2189 | 1897 | 292 | 72.54 | 2.35-2.40 | 2223 | 1945 | 278 | 66.45 | 2.45-2.50 |
| Harvesting cost (Rs/acre) | 2643 | 2765 | -122 | 88.45 | 1.95-2.00 | 2700 | 2798 | -98 | 72.76 | 2.35-2.40 | 2678 | 2803 | -125 | 75.49 | 2.25-2.30 |
| Post-harvest cost (Rs/acre) | 7569 | 7816 | -247 | 78.15 | 3.25-3.30 | 7403 | 7789 | -386 | 87.12 | 2.45-2.50 | 7600 | 7892 | -292 | 98.33 | 2.55-2.60 |
| Total labor (days/acre) | 28.94 | 36.71 | -7.77 | 1.2 | 2.25-2.30 | 28.16 | 36.18 | -8.02 | 1.32 | 2.35-2.40 | 28.6 | 35.73 | -7.13 | 1.05 | 2.15-2.20 |
| Family labor (days/acre) | 12.61 | 14.67 | -2.06 | 0.38 | 2.55-2.60 | 13.18 | 14.77 | -1.59 | 0.45 | 2.35-2.40 | 12.81 | 14.23 | -1.42 | 0.34 | 2.25-2.30 |
| Total cost (Rs/acre) | 21883 | 26751 | -4868 | 455.78 | 2.30-2.35 | 22101 | 27203 | -5102 | 481.23 | 2.35-2.40 | 22345 | 27504 | -5159 | 563.23 | 2.40-2.45 |
| Rice yield (kg/acre) | 2644 | 2548 | 96 | 27.19 | 2.55-2.60 | 2685 | 2596 | 89 | 32.41 | 2.15-2.20 | 2703 | 2611 | 92 | 32.16 | 2.35-2.40 |
| Income from rice (Rs/acre) | 39917 | 35010 | 4907 | 448.18 | 2.40-2.45 | 40448 | 35119 | 5329 | 401.34 | 2.10-2.15 | 39387 | 34596 | 4791 | 423.18 | 2.35-2.40 |
