# Supplementary material for: Economic suitability of direct seeded rice across different geographies in India
Source: PLoS One. 2025 Apr 18;20(4):e0321472. doi: 10.1371/journal.pone.0321472 (PMC12007715; doi:10.1371/journal.pone.0321472)
Supplement: S4 Table — Effect of adopting DSR practices on income, production, and expenses associated with paddy cultivation in Telangana. (DOCX) [file pone.0321472.s004.docx]

**Table S4**. Effect of adopting DSR practices on income, production, and expenses associated with paddy cultivation in Telangana

|  | **NNM** | | | | | **KBM** | | | | | **RM** | | | | |
| --- | --- | --- | --- | --- | --- | --- | --- | --- | --- | --- | --- | --- | --- | --- | --- |
|  | **DSR adopters** | **DSR non-adopters** | **ATT** | **SE** | **Critical level of hidden bias** | **DSR adopters** | **DSR non-adopters** | **ATT** | **SE** | **Critical level of hidden bias** | **DSR adopters** | **DSR non-adopters** | **ATT** | **SE** | **Critical level of hidden bias** |
| Land preparation cost (Rs/acre) | 707 | 4235 | -3528 | 122.18 | 2.45-2.50 | 756 | 4215 | -3459 | 143.87 | 2.70-2.75 | 781 | 4235 | -3454 | 118.19 | 2.55-2.60 |
| Seed and seed treatment cost (Rs/acre) | 856 | 1345 | -489 | 54.89 | 2.65-2.70 | 847 | 1381 | -534 | 56.19 | 2.35-2.40 | 817 | 1342 | -525 | 58.39 | 2.70-2.75 |
| Crop establishment cost (Rs/acre) | 958 | 2987 | -2029 | 143.87 | 2.35-2.40 | 978 | 2899 | -1921 | 121.34 | 2.35-2.40 | 966 | 2871 | -1905 | 155.32 | 2.35-2.40 |
| Total fertilizer cost (Rs/acre) | 3456 | 3981 | -525 | 67.19 | 2.55-2.60 | 3478 | 3998 | -520 | 69.45 | 2.55-2.60 | 3434 | 3978 | -544 | 65.21 | 2.55-2.60 |
| Irrigation cost (Rs/acre) | 1718 | 2915 | -1197 | 121.34 | 1.95-2.00 | 1745 | 2938 | -1193 | 111.32 | 2.45-2.50 | 1724 | 2944 | -1220 | 144.76 | 2.15-2.20 |
| Weed control cost (Rs/acre) | 2097 | 1781 | 316 | 65.77 | 2.15-2.20 | 2125 | 1798 | 327 | 56.78 | 2.15-2.20 | 2133 | 1809 | 324 | 60.12 | 2.45-2.50 |
| Pest control cost (Rs/acre) | 1977 | 1789 | 188 | 71.83 | 2.35-2.40 | 1987 | 1795 | 192 | 65.87 | 2.65-2.70 | 2003 | 1762 | 241 | 66.19 | 2.55-2.60 |
| Harvesting cost (Rs/acre) | 1987 | 2261 | -274 | 45.87 | 2.15-2.20 | 2001 | 2345 | -344 | 39.67 | 2.35-2.40 | 1987 | 2322 | -335 | 42.98 | 2.65-2.70 |
| Post-harvest cost (Rs/acre) | 3531 | 4261 | -730 | 154.12 | 2.55-2.60 | 3578 | 4298 | -720 | 166.17 | 2.45-2.50 | 3561 | 4277 | -716 | 150.32 | 2.55-2.60 |
| Total labor (days/acre) | 33.27 | 40.01 | -6.74 | 0.45 | 2.65-2.70 | 33.11 | 41.83 | -8.72 | 0.37 | 2.55-2.60 | 33.09 | 40.73 | -7.64 | 0.38 | 2.15-2.20 |
| Family labor (days/acre) | 12.78 | 15.23 | -2.45 | 0.27 | 2.70-2.75 | 12.44 | 15.26 | -2.82 | 0.31 | 2.15-2.20 | 12.32 | 15.113 | -2.793 | 0.25 | 1.95-2.00 |
| Total cost (Rs/acre) | 17654 | 25423 | -7769 | 432.17 | 2.35-2.40 | 17881 | 25502 | -7621 | 446.65 | 2.55-2.60 | 17654 | 25417 | -7763 | 430.3 | 2.65-2.70 |
| Rice yield (kg/acre) | 2532 | 2389 | 143 | 33.26 | 2.25-2.30 | 2541 | 2425 | 116 | 41.27 | 2.35-2.40 | 2549 | 2431 | 118 | 34.52 | 2.35-2.40 |
| Income from rice (Rs/acre) | 41322 | 33244 | 8078 | 1256 | 2.15-2.20 | 42178 | 34122 | 8056 | 1345 | 2.70-2.75 | 42456 | 34189 | 8267 | 1280 | 2.45-2.50 |
