# Supplementary material for: Economic suitability of direct seeded rice across different geographies in India
Source: PLoS One. 2025 Apr 18;20(4):e0321472. doi: 10.1371/journal.pone.0321472 (PMC12007715; doi:10.1371/journal.pone.0321472)
Supplement: Attachment — Submitted filename: 04 DEC 2024 REVIEWED MS PONE-D-24-42084.pdf [file pone.0321472.s005.pdf]

PLOS ONE

Economic Suitability of Direct Seeded Rice across Different Geographies in India

--Manuscript Draft--

**Manuscript Number:**	PONE-D-24-42084
**Article Type:**	Research Article
**Full Title:**	Economic Suitability of Direct Seeded Rice across Different Geographies in India
**Short Title:**	Economic Suitability of DSR
**Corresponding Author:**	Kumar Abbhishek, Ph.D.<br>Dr. Reddy's Foundation<br>Hyderabad, Telangana INDIA
**Keywords:**	Economic suitability, Impact assessment, Dry-Direct Seeded Rice, Puddled Transplanted rice, Agro-climatic zones, Propensity Score Matching approach, Endogenous switch regression method
**Abstract:**	Puddled transplanted rice (PTR) is being replaced by dry direct-seeded rice (dDSR) to address manpower, water, and agricultural costs. The economic suitability of dDSR in different agro-climatic regions limits its widespread adoption. We use plot and household data to estimate the impact of dDSR adoption in four Indian rice-growing states. We first used propensity score matching (PSM) to assess how dDSR adoption affected operation-wise cultivation costs, paddy yield, and net income. The yield effect on DSR adoption was estimated using endogenous switch regression (ESR) to account for observed and unobserved heterogeneity. Both PSM and ESR-based results show that DSR adoption may increase paddy yield in Uttar Pradesh, Andhra Pradesh, and Telangana and decrease it in Madhya Pradesh, but net income from paddy farming increased significantly (Rs5009/acre to 8134 based on different locations) in all four states. Adopting dDSR helps resource-poor Indian farmers reduce paddy production costs and increase income. Therefore, Central and State governments must implement policies and strategies to encourage non-adopters to adopt dDSR.
**Order of Authors:**	Shiladitya Dey, Ph.D.
	Kumar Abbhishek, Ph.D.
	Suman Saraswathibatla, MA
	Debabrata Das, Ph.D.
**Additional Information:**	
**Question**	**Response**
**Financial Disclosure**<br><br>Enter a financial disclosure statement that describes the sources of funding for the work included in this submission. Review the submission guidelines for detailed requirements. View published research articles from *PLOS ONE* for specific examples.<br><br>This statement is required for submission and **will appear in the published article** if the submission is accepted. Please make sure it is accurate.	The author(s) received no specific funding for this work.

Powered by Editorial Manager® and ProduXion Manager® from Aries Systems Corporation

**Funded studies**<br>Enter a statement with the following details:<br>• Initials of the authors who received each award<br>• Grant numbers awarded to each author<br>• The full name of each funder<br>• URL of each funder website<br>• Did the sponsors or funders play any role in the study design, data collection and analysis, decision to publish, or preparation of the manuscript?<br><br>Did you receive funding for this work?	
**Competing Interests**<br><br>Use the instructions below to enter a competing interest statement for this submission. On behalf of all authors, disclose any competing interests that could be perceived to bias this work—acknowledging all financial support and any other relevant financial or non-financial competing interests.<br><br>This statement is required for submission and **will appear in the published article** if the submission is accepted. Please make sure it is accurate and that any funding sources listed in your Funding Information later in the submission form are also declared in your Financial Disclosure statement.<br><br>View published research articles from *PLOS ONE* for specific examples.	The authors have declared that no competing interests exist.

Powered by Editorial Manager® and ProduXion Manager® from Aries Systems Corporation

**NO authors have competing interests**<br><br>Enter: *The authors have declared that no competing interests exist.*<br><br>**Authors with competing interests**<br><br>Enter competing interest details beginning with this statement:<br><br>*I have read the journal's policy and the authors of this manuscript have the following competing interests: [insert competing interests here]*<br><br>* typeset	
**Ethics Statement**<br><br>Enter an ethics statement for this submission. This statement is required if the study involved:<br><br>• Human participants<br>• Human specimens or tissue<br>• Vertebrate animals or cephalopods<br>• Vertebrate embryos or tissues<br>• Field research<br><br>Write "N/A" if the submission does not require an ethics statement.<br><br>General guidance is provided below. Consult the submission guidelines for detailed instructions. **Make sure that all information entered here is included in the Methods section of the manuscript.**	Face-to-face questionnaire surveys were implemented at the participants' residences or fields to collect the data. Verbal consent was obtained from all respondents before the interview. Verbal consent was obtained for two reasons: First, it was anticipated that a substantial number of the respondents would be illiterate and apprehensive about signing consent documents. Secondly, the socioeconomic survey did not collect biological data, tissues, or human samples for clinical trials. The interviewers clarified the procedure and emphasized that the subjects' participation was voluntary and that the data collected would be used for research purposes.

Powered by Editorial Manager® and ProduXion Manager® from Aries Systems Corporation

**Format for specific study types**<br><br>**Human Subject Research (involving human participants and/or tissue)**<br>• Give the name of the institutional review board or ethics committee that approved the study<br>• Include the approval number and/or a statement indicating approval of this research<br>• Indicate the form of consent obtained (written/oral) or the reason that consent was not obtained (e.g. the data were analyzed anonymously)<br><br>**Animal Research (involving vertebrate animals, embryos or tissues)**<br>• Provide the name of the Institutional Animal Care and Use Committee (IACUC) or other relevant ethics board that reviewed the study protocol, and indicate whether they approved this research or granted a formal waiver of ethical approval<br>• Include an approval number if one was obtained<br>• If the study involved *non-human primates*, add *additional details* about animal welfare and steps taken to ameliorate suffering<br>• If anesthesia, euthanasia, or any kind of animal sacrifice is part of the study, include briefly which substances and/or methods were applied<br><br>**Field Research**<br><br>Include the following details if this study involves the collection of plant, animal, or other materials from a natural setting:<br>• Field permit number<br>• Name of the institution or relevant body that granted permission	
**Data Availability**<br><br>Authors are required to make all data underlying the findings described fully available, without restriction, and from the time of publication. PLOS allows rare exceptions to address legal and ethical concerns. See the PLOS Data Policy and FAQ for detailed information.	Yes - all data are fully available without restriction

Powered by Editorial Manager® and ProduXion Manager® from Aries Systems Corporation

A Data Availability Statement describing where the data can be found is required at submission. Your answers to this question constitute the Data Availability Statement and **will be published in the article**, if accepted.<br><br>**Important:** Stating 'data available on request from the author' is not sufficient. If your data are only available upon request, select 'No' for the first question and explain your exceptional situation in the text box.<br><br>Do the authors confirm that all data underlying the findings described in their manuscript are fully available without restriction?	
**Describe where the data may be found in full sentences. If you are copying our sample text, replace any instances of XXX with the appropriate details.**<br><br>• If the data are **held or will be held in a public repository**, include URLs, accession numbers or DOIs. If this information will only be available after acceptance, indicate this by ticking the box below. For example: *All XXX files are available from the XXX database (accession number(s) XXX, XXX.).*<br>• If the data are all contained **within the manuscript and/or Supporting Information files**, enter the following: *All relevant data are within the manuscript and its Supporting Information files.*<br>• If neither of these applies but you are able to provide **details of access elsewhere**, with or without limitations, please do so. For example:<br><br>*Data cannot be shared publicly because of [XXX]. Data are available from the XXX Institutional Data Access / Ethics Committee (contact via XXX) for researchers who meet the criteria for access to confidential data.*<br><br>*The data underlying the results presented in the study are available from (include the name of the third party*	All relevant data are within the manuscript and its Supporting Information files.

and contact information or URL).<br>• This text is appropriate if the data are owned by a third party and authors do not have permission to share the data.<br><br>* typeset	
Additional data availability information:	

Manuscript Click here to access/download;Manuscript;Manuscript.docx

Economic Suitability of Direct Seeded Rice across Different Geographies in India

Shiladitya Dey[1#] , Kumar Abbhishek[1*#] , Suman Saraswathibatla[1] , Debabrata Das[4]

[1] Action for Climate and Environment, Dr. Reddy's Foundation, Hyderabad, Telangana, India

* Corresponding author

E-mail: abbhishek.k@drreddysfoundation.org

# These authors contributed equally to this work

Abstract

Puddled transplanted rice (PTR) is being replaced by dry direct-seeded rice (dDSR) to address manpower, water, and agricultural costs. The economic suitability of dDSR in different agro-climatic regions limits its widespread adoption. We use plot and household data to estimate the impact of dDSR adoption in four Indian rice-growing states. We first used propensity score matching (PSM) to assess how dDSR adoption affected operation-wise cultivation costs, paddy yield, and net income. The yield effect on DSR adoption was estimated using endogenous switch regression (ESR) to account for observed and unobserved heterogeneity. Both PSM and ESR-based results show that DSR adoption may increase paddy yield in Uttar Pradesh, Andhra Pradesh, and Telangana and decrease it in Madhya Pradesh, but net income from paddy farming increased significantly (Rs5009/acre to 8134 based on different locations) in all four states. Adopting dDSR helps resource-poor Indian farmers reduce paddy production costs and increase income. Therefore, Central and State governments must implement policies and strategies to encourage non-adopters to adopt dDSR.

Keywords: Economic suitability, Impact assessment, Dry-Direct Seeded Rice, Puddled Transplanted rice, Agro-climatic zones, Propensity Score Matching approach, Endogenous switch regression method

1. Introduction

Rice is vital to global food security because half the world eats it [1]. India was first in the paddy area and second in production. India produces 21% of global rice [2]. Global demand for milled rice is projected to increase by 96 million tonnes by 2040 compared to 2015 [3]. Rice production must rise for food security and sustainability. Increased rice production must be environmentally friendly (using less water, labor, and agrochemicals). This can be achieved by closing rice yield gaps and preserving natural resources, especially in India's Indo-Gangetic Plane and Southern States.

Groundwater is used extensively in conventional paddy cultivation. PTR involves physically transplanting paddy into tilled and puddled soil. Evaporation and percolation use 60–80% more water than crops need, raising paddy cultivation costs [4, 5]. Water-efficient, sustainable, cost-effective paddy farming requires water conservation [6]. Dry-direct seeded rice (dDSR) saves water in paddy production [6]. Paddy seeds are directly sown in the main field and established by pre-monsoon rainfall in dDSR. DSR in non-puddled soils saved 35-57% of Northwest India's water compared to PTR [7]. Most research shows that dDSR reduces paddy water use. The amount varies greatly by soil type. More importantly, water reduction lowers dDSR yield [8, 9]. Therefore, it is crucial to determine whether dDSR adoption is economically viable in different geographies with different soil types and properties. DSR productivity is often lower than PTR due to panicle sterility, poor crop stand, micronutrient deficiencies, higher weed pressure, and root-knot nematodes. Research indicates that DSR yields 3 t ha−1 more grain than PTR (2 t ha−1) due to higher panicle number, 1000 kernel weight, and lower sterility percentage [10, 11]. Table 1 summarises the contrary findings and shows that DSR may or may not yield better than PTR in similar rice ecologies. Thus, understanding whether DSR is economically and yield-wise suitable in different regions is crucial.

Table 1. Paddy yield comparison in DSR and PTR

Author(s)	Country	Rice ecology	PTR (t ha^{-1})	DSR (t ha^{-1})
Hobbs et al. (2002).	India and Nepal	Favorable irrigated	5.30	5.40
Sharma et al. (2004).	India	Favorable irrigated	5.22	5.99
Sarkar et al. (2003).	India	Rainfed low land	2.99	3.15
Rath et al. (2000).	India	Rainfed lowland	4.17	4.64
Tripathi et al. (2005a)	India	Rainfed lowland	6.35	6.09
Singh et al. (2009a).	India	Rainfed lowland	6.8	6.6

Source: Authors' compilation

The performance-based effectiveness of dDSR has been reported in various Indian agro-climatic zones [12]. In rice-growing ecologies, dDSR is also an attractive technology [13], but soil type, water management, and climate may limit its performance [14-16]. Most studies compared PTR and DSR irrigation water savings and yield using experimental plots. Some studies found that long-term DSR use changes soil chemical and physical properties [17-19]. Few studies used DSR and PTR to compare greenhouse gas emissions and global warming potential [20-22]. Rarely do studies determine whether resource-poor small and medium farmers can profit from DSR over PTR and, more importantly, whether DSR is economically equally suitable in different ago-climatic conditions. This study evaluates DSR establishment methods in India's four rice-growing agro-climatic zones. Our plot-level data comes from Uttar Pradesh (UP), Madhya Pradesh (MP), Andhra Pradesh (AP), and Telangana (TS). This study adds three things to the literature. The study uses farm-level survey data to determine how DSR affects paddy yield, operation-wise cost of cultivation, and net income. Most research uses field trial data, which may not accurately reflect paddy farmers' conditions [23-26]. Second, endogenous switching regression accounts for observed and unobserved heterogeneity [27]. Third, the study evaluates DSR's economic viability using yield, income, and cultivation costs for four agro-climatic zones. Our literature review found no previous analysis of the DSR's economic impact using survey data from four states in different agro-climatic conditions.

2. Methodology

2.1. Propensity score matching method

The decision to adopt the DSR method is a binary choice for the farmer. Farmers will use the technology if the net gain from adopting DSR is greater than not adopting it. The farmers' net income from adopting DSR compared to non-adoption is represented as K^* (Equation (1). If $K^* > 0$ indicates that the farmer's net gain from adopting DSR is greater than the benefit from not adopting it. However, K^* is not possible to be measured directly. Nonetheless, it can be expressed as a function of quantifiable components inside the latent variable model described below:

$$K^* = \alpha Z_i + \varepsilon_{i,} \quad K_i = 1[K_i^* > 0], \tag{1}$$

where K_i is a binary variable equal to 1 if the household has adopted the dDSR and 0 if it has not. The coefficient α represents the parameters that need to be measured. Z_i represents the characteristics of households and farms in vector form. The error term ε_i is assumed to follow a normal distribution. Equation (2) illustrates the probability of implementing the DSR establishment method:

$$\Pr(K_i = 1) = \Pr(K_i^* > 0) = \Pr(\varepsilon_i < \alpha Z_i) = 1 - G(-\alpha Z_i) \tag{2}$$

Here, G represents the cumulative distribution function for ε_i. Regression models such as logit and probit are typically derived based on the assumptions about the functional form of G. Various socioeconomic and demographic parameters, yield, and net revenue from production are expected to influence the adoption of DSR technology. In order to establish a connection between the decision to adopt DSR and the possible consequences of adoption, we will examine a risk-neutral production system that aims to maximize net return ∋. This system operates in a competitive market for both output and input and utilizes a single-output technology that is quasi-concave in the vector of variable inputs, H. This can be expressed as equation (3):

$$max \ni = CP(H, Z) - LH \quad (3)$$

R is the selling price of the paddy, P is the expected production level, L is a common vector for input costs, and H describes the attributes and features of households and farms. The net return from adopting DSR can be represented as a mathematical function of the choice of DSR adoption K, the output price, input variables, and household features, as shown in Equation (4).

$$max \ni = \ni (K, L, R, Z) \quad (4)$$

Equations 4 suggest that the decision to adopt DSR technology, output and input pricing, and household and farm characteristics can affect input demand, net return, and farm productivity.

The PSM method can offer an impartial evaluation of the impact of the treatment (i.e., adoption of DSR), provided that the outcomes are not influenced by the assignment to treatment, given the pre-treatment baseline covariates. The PSM approach primarily quantifies the impact of the treatment on the population that received it, as represented by equation (5).

$$\forall|_{K=1} = T(\forall |\ K = 1) = T(V_1 \mid 1) - T(V_0 \mid 1) \quad (5)$$

Where $\forall$ is the average treatment effect on treated (ATT), V_1 indicates the outcome values of the DSR adopters, and V_0 is the value of the same variables for non-adopters. However, the study does not estimate $T(V_0 \mid K = 1)$. Instead, the study measures the difference ($\forall^e$) between $T(V_1 \mid K = 1)$ and $T(V_0 \mid K = 1)$. Hence, $\forall^e$ acts as a potential bias estimator.

The PSM approach can mitigate the sample selection bias when experimental and/or panel data are unavailable [28]. The PSM model uses conditional probability to determine the likelihood of farmers adopting DSR technology depending on their pre-adoption characteristics [29]. The PSM model relies on the unconfoundedness assumption, also known as the conditional independence assumption. This assumption implies that, after controlling for E, DSR technology adoption is random and unrelated to the outcome variables. PSM can be represented by Equation 6.

$$p(E) = \Pr\{K = 1 \mid E\} = T\{K \mid E\} \quad (6)$$

Where $K = \{0,1\}$ is the indicator for DSR adoption, and E is the pre-adoption attributes. The conditional distribution of E in given $p(E)$ is the same in both DSR adopters and non-adopters.

The PSM technique does not necessitate making assumptions about the functional form, which involves describing the relationship between outcomes and predictors of outcomes. The primary limitation of the PSM technique is the assumption of unconfoundedness. Even after conditioning, there may still be systemic variations in the outcomes of non-adopters and adopters due to unmeasured baseline variables. However, the PSM technique allows checking the specification to eliminate biases that exceed the average.

Once the propensity score (PS) has been established, the average treatment effect on the treated (ATT) can be calculated using equation 7:

$$\forall = T\{(V_1 - V_0 \mid K = 1)\} = T\{T(V_1 - V_0 \mid K = 1), p(E)\} = T[T\{(V_1 \mid K = 1), p(E)\}] - [T\{(V_1 \mid K = 0), p(E)\}] \quad (7)$$

Various matching strategies can be employed to match adopters with non-adopters who have similar PS. This study utilizes nearest neighbor matching (NNM), kernel-based matching (KBM), and radius matching (RM) to assess the reliability of the results.

2.2. Endogenous switch regression approach

PSM method reduces selection bias caused by observables. However, it does not affect selection bias produced by the unobservable. The PSM method limits conclusions to individuals or farmers whose attributes are present in both the sample and control groups. In this study, we aim to measure the effects of the adoption of DSR over PTR methods on costs, productivity, and income through cross-sectional household data. It is crucial to mention that, like in past research, in this study, the treatment and control groups are not randomly allocated [30]. Smallholder households may self-select into the treatment group by adopting DSR, an

example of endogeneity and self-selection. The second concern is estimating the impact of the adoption of DSR through an empirical method. However, in our case, we anticipate that household and farm characteristics may affect DSR's adoption, which further influences outcome variables.

The Endogenous Switch Regression (ESR) model can overcome the abovementioned concerns associated with impact assessment. The ESR model treats the adoption of DSR as a regime shifter. Moreover, ESR considers the observed and unobserved variations among smallholders in the two adoption schemes. Two steps make up the ESR regime. An initial step is a binary choice criterion function or selection equation. In such a situation, the smallholder will evaluate the available resources and management alternatives before deciding whether to adopt DSR. The farmer matches the expected utility of DSR adoption, $D_{i,DSR}^{*}$, to the expected utility of PTR ($D_{i,PTR,}^{*}$ or Conventional method). Farmers will adopt DSR if $D_{i,DSR}^{*} > D_{i,PTR}^{*}$ and will not adopt if $D_{i,DSR}^{*} < D_{i,PTR}^{*}$. D_i^* is the adoption dummy variable that is unobservable, but we do observe D_i. Initially, we will estimate with probit (using Equation 8)

$$D_i^* = M_i \partial + \cup_i \text{ with } \begin{cases} 1\ if\ D_{i,DSR}^{*} > D_{i,PTR}^{*} \\ 0\ if\ D_{i,DSR}^{*} < D_{i,PTR}^{*} \end{cases} \quad (8)$$

The vectors A_i include farm and farmer's socioeconomic and demographic attributes, ∂ is the vector of parameters to be measured, $\cup_i$ is a random error. In the following step, using the findings of the criterion function, we specify two regime equations that explain the outcome variable of interest. The link between a vector of explanatory variables W and the outcome P may be expressed as $P = f(W)$. More precisely, there are two distinct regimes (Equation 9):

$$Regime\ 1: P_{i,DSR} = W_i\gamma + \rho_{i,DSR,} \quad if\ D_i = 1$$

$$Regime\ 1: P_{i,PTR} = W_i\delta + \rho_{i,PTR,} \quad if\ D_i = 0 \quad (9)$$

Where γ and δ are parameters to be measured. Moreover, variables under M_i and W_i are allowed to overlap, and error terms $\cup_i, \rho_{i,DSR,}$ and $\rho_{i,PTR}$ have tri-variate normal distribution with zero mean and non-singular covariate matrix [31]. The absence of simultaneous observation of Regime 1 and Regime 2 results in an undefined covariance between $\rho_{i,DSR}$, and $\rho_{i,PTR}$. Furthermore, the expected values of $\rho_{i,DSR}$, and $\rho_{i,PTR}$ are not zero due to the correlation between the error term of the selection Equation 8. We assume that $\Delta_{\cup}^2 = 1$ (∂ is estimable only up to scalar). Δ_{DSR}^2 and Δ_{PTR}^2 are variances of the disturbance term used in Equation 9. Moreover, $\Delta_{\cup\rho DSR}$ and $\Delta_{\cup\rho PTR}$ are covariance of $\cup_i$ and $\rho_{i,DSR}$ and covariance of $\cup_i$ and $\rho_{i,PTR}$, respectively. The correlation between the error terms in the selection Equation (8) and the regime Equation (9), which are assessed as truncated error terms, causes the expected values of the error terms in Equation (9) to be non-zero [31]. The expected value is calculated as the product of the variance and Inverse Mills Ratios (IMRs) assessed at M_i^{∂}, denoted as $\aleph_{DSR}$ and $\aleph_{PTR}$, respectively, in Equations 10 and 11.

$$A = \left(p_{i,DSR} \mid D = 1\right) = \Delta_{\cup\rho\, DSR} \frac{\varphi\left(\frac{M_{\partial}^{i}}{\Delta_{\cup}^{2}}\right)}{\vartheta\left(\frac{M_{\partial}^{i}}{\Delta_{\cup}^{2}}\right)} = \Delta_{\cup\rho DSR}\, \aleph_{DSR} \tag{10}$$

$$A = \left(p_{i,PTR} \mid D = 0\right) = \Delta_{\cup\rho\, PTR} \frac{\varphi\left(\frac{M_{\partial}^{i}}{\Delta_{\cup}^{2}}\right)}{\vartheta\left(\frac{M_{\partial}^{i}}{\Delta_{\cup}^{2}}\right)} = \Delta_{\cup\rho PTR}\, \aleph_{\rho PTR} \tag{11}$$

The ESR model can be implemented using a two-stage approach, where regime equations incorporate IMRs. In our investigation, we employ the full information maximum likelihood technique described by Lokshin & Sajaia [32]. For the ESR model to be accurately stated, the factors influencing the selection equation (Equation 8) must include at least one instrument. Additionally, the factors influencing the outcome variables in equation (Equation 9) should be correlated with the adoption of DSR but not directly correlated with the outcome variables. The ESR model's conditional expectations compare the expected outcome variables of DSR

adopters and non-adopters in four hypothetical counterfactual circumstances. These cases involve scenarios where adopter smallholders did not adopt, and non-adopter smallholders did adopt DSR. According to Noltze et al. [33], the following four scenarios can be summarized as:

DSR households with adoption (observed):

$$A(P_{DSR} \mid D = 1) = W'\gamma + \Delta_{\cup\rho DSR} \aleph_{\rho DSR} \quad (12a)$$

DSR households without DSR adoption (counterfactual):

$$A(P_{PTR} \mid D = 1) = W'\delta + \Delta_{\cup\rho PTR} \aleph_{\rho DSR} \quad (12b)$$

PTR households without adoption (observed):

$$A(P_{PTR} \mid D = 0) = W'\delta + \Delta_{\cup\rho PTR} \aleph_{\rho PTR} \quad (12c)$$

PTR households with adoption (counterfactual):

$$A(P_{DSR} \mid D = 0) = W'\gamma + \Delta_{\cup\rho DSR} \aleph_{\rho PTR} \quad (12d)$$

In addition to the marginal impacts of W on crop yield and cost of cultivation, we are aiming to determine the treatment impact of DSR adoption. According to Greene [30], Fuglie and Bosch [31], and Alene and Manyong [34] equations 12(a)–12(b) can be utilized to calculate the overall impact of adopting DSR (average treatment effect on the treated, or ATT) and the average treatment effects on those who did not adopt DSR, i.e., the average treatment effect on untreated (ATU). Moreover, we can obtain ATT and ATU from Equation 13 and Equation 14.

$$ATT = A(P_{DSR} \mid D = 1) - A(P_{PTR} \mid D = 1) \quad (13)$$

$$ATU = A(P_{DSR} \mid D = 0) - AA(P_{PTR} \mid D = 0) \quad (14)$$

2.3. Ethics statement

Face-to-face questionnaire surveys were implemented at the participants' residences or fields to collect the data. Verbal consent was obtained from all respondents before the interview. Verbal consent was obtained for two reasons: First, it was anticipated that a substantial number of the respondents would be illiterate and apprehensive about signing consent documents. Secondly, the socioeconomic survey did not collect any biological data or tissues or human samples for clinical trial. The interviewers clarified the procedure and emphasized that the subjects' participation was voluntary and that the data collected would be used for research purposes.

2.4. Data

The data used in this study is derived from household surveys of farmers conducted in UP, MP, AP, and TS, four major rice-producing states in India. In these states, labor availability is among the lowest compared to other states, so the DSR method may prove viable and practical. A multi-stage sampling procedure (from state to district to village to farmers) was implemented for the face-to-face questionnaire survey. A survey was conducted on 537 farmers from four different states. Certain surveyed farmers implemented DSR and PTR on distinct plots, whereas others exclusively utilized DSR or PTR on their respective plots. We take each farm or plot as a separate establishment for the analysis done in the paper. In UP, there were 155 DSR plots and 197 PTR plots; in MP, there were 162 DSR plots and 141 PTR plots; in AP, there were 133 DSR plots and 217 PTR plots; while in TS, there were 197 DSR plots and 205 PTR plots. Our plot-level analysis for yield and cost estimation has 1407 observations. Figure 1 shows that, across the location, among the surveyed households, 46.5-56% of farmers have adopted the DSR practice for producing paddy in the Kharif season.

3. Results and Discussion

3.1. Descriptive statistics

Table 2 shows descriptive statistics for socioeconomic variables. The results show that higher education, access to extension services (like agricultural officer/expert visits, agricultural university and Krishi Vigyan Kendra and Krishi Mela services), and community organization membership promote DSR adoption in all four states, regardless of age. However, if irrigation is not a problem, farmers choose PTR. Farmers may prefer DSR over PTR due to lower cultivation costs, but more weed pressure and irrigation frequency detract from its appeal.

Table 2. Descriptive statistics and mean comparison of DSR and PTR in four different states in India

	Uttar Pradesh					Madhya Pradesh					Andhra Pradesh					Telangana				
	DSR		PTR		p-value	DSR		PTR		p-value	DSR		PTR		p-value	DSR		PTR		p-value
Socioeconomic variables	**Mean**	**Std. Dev**	**Mean**	**Std. Dev**		**Mean**	**Std. Dev**	**Mean**	**Std. Dev**		**Mean**	**Std. Dev**	**Mean**	**Std. Dev**		**Mean**	**Std. Dev**	**Mean**	**Std. Dev**	
Age of household head	42.61	14.26	44.29	14.11	0.191	39.71	12.54	41.13	14.31	0.142	45.33	15.82	46.64	15.41	0.116	44.31	11.16	43.12	12.36	0.162
Education level (Years)	8.43	2.27	6.12	2.21	0.131	9.11	317	7.89	4.19	0.243	10.53	2.23	8.17	1.92	0.088	11.42	2.83	9.76	3.23	0.082
Household size	5.17	2.04	5.32	2.11	0.224	4.89	1.87	5.33	2.19	0.173	4.47	1.73	4.75	1.89	0.153	4.33	1.58	5.73	1.55	0.123
Community organization(s) member (%)	27.13	4.18	22.56	3.28	0.162	33.17	3.43	26.19	3.11	0.093	44.72	4.19	33.17	3.77	0.083	48.13	5.54	39.37	5.18	0.092
Farm experience	21.24	6.13	23.19	5.11	0.256	18.39	6.87	20.76	5.76	0.202	21.85	6.11	22.06	7.23	0.311	22.33	5.56	23.01	6.34	0.256
Assured irrigation	28.37	5.17	42.17	6.92	0.042	22.39	5.89	36.17	6.15	0.073	44.18	7.13	57.19	8.71	0.074	66.15	8.17	83.12	8.33	9.46
Livestock ownership (%)	0.64	0.18	0.68	0.16	0.222	0.58	0.17	0.6	0.14	0.188	0.61	0.22	0.67	0.19	0.223	0.48	0.17	0.5	0.21	0.187
Smartphone ownership	0.66	0.18	0.57	0.11	0.112	0.63	0.23	0.58	0.17	0.222	0.75	0.11	0.72	0.13	0.232	0.78	0.15	0.72	0.13	0.183
Extension service	18.42	3.67	14.12	3.51	0.184	21.73	4.66	17.82	3.47	0.215	36.58	5.22	31.82	4.51	0.193	39.16	5.72	32.36	3.95	0.129
Crop insurance	12.79	2.46	11.87	2.65	0.246	15.16	2.37	14.63	2.56	0.261	17.97	3.27	15.83	3.27	0.213	19.77	3.45	17.46	2.89	0.221
Total landholding (acre)	1.63	0.42	1.69	0.39	0.154	2.43	0.54	2.51	0.51	0.243	1.73	0.83	1.68	0.73	0.202	2.46	1.02	2.54	0.93	0.197
Outcome variables																				
Land preparation cost (Rs/acre)	556	83.23	3606	346.17	0.014	876	103.72	2634	264.83	0.012	777	123.92	2402	279.35	0.008	669	92.37	5040	456.81	0.004
Seed and seed treatment cost	959	58.42	1248	76.39	0.043	1017	114.57	2510	254.39	0.032	866	115.23	1397	165.76	0.036	827	89.17	1329	145.74	0.027
Sowing/Transplanting cost	1151	231.54	2773	335.83	0.004	1498	245.91	5198	554.76	0.001	1445	234.17	3615	337.89	0.003	945	465.46	3563	654.17	0.002
Total fertilizer cost (Rs/acre)	2286	321.16	2607	455.83	0.137	2776	355.18	3678	448.27	0.082	3155	376.19	3113	418.62	0.167	3854	444.17	4261	537.87	0.143

Irrigation cost (Rs/acre)	1754	273.67	2348	389.45	0.112	816	289.14	1549	333.54	0.054	1768	334.28	2715	476.17	0.065	1715	287.34	2874	322.19	0.043
Weed control cost (Rs/acre)	1646	209.17	1478	188.76	0.167	1432	333.18	1235	342.18	0.152	1599	245.19	1781	311.54	0.132	2054	198.62	1834	202.41	0.156
Pest control cost	1680	265.16	1297	176.54	0.108	1216	265.17	1052	211.45	0.167	2245	372.74	1879	187.54	0.034	2000	244.87	1763	221.34	0.109
Harvesting cost	2490	333.87	2496	287.51	0.243	1763	436.73	1800	387.19	0.237	2774	224.45	2759	276.38	0.221	1542	278.87	2346	299.16	0.067
Post-harvest cost	6426	1176	6872	1380	0.187	5992	1083	6332	1265.34	0.143	7592	1065	7894	1285	0.254	3037	556.87	4144	455.9	0.081
Total labor (days/acre)	27.16	3.67	35.15	4.76	0.052	28.31	4.87	35.18	5.87	0.243	28.55	3.45	36.16	4.27	0.057	33.18	2.34	36.16	2.76	0.116
Family labor (days/acre)	14.13	2.64	16.67	3.08	0.176	13.28	3.17	16.69	3.18	0.183	12.77	2.18	14.21	1.98	0.132	13.33	1.83	15.75	1.65	0.211
Total cost (Rs/acre)	18948	2189	24725	3546	0.043	17386	4567	25988	5432	0.035	22221	3786	27555	4111	0.069	16643	3546	27154	4467	0.005
Rice yield (kg/acre)	1793	246.87	1739	254.19	0.219	1777	143	2043	154	0.046	2666	146	2523	231	0.443	2523	118	2371	107	0.172
Income from rice (Rs/acre)	20193	3467	13237	3142	0.062	22241	4653	19162	4576	0.082	40217	4776	34883	3987	0.072	48955	5671	33306	4782	0.001
Rice selling price (Rs/Quintal)	2183	45.64	2183	38.65	0.165	2230	35.76	2210	45.82	0.215	2342	28.97	2342	32.45	0.177	2600	26.8	2550	33.18	0.119

No matter the location, the DSR method (Rs.719.5/acre) costs 79% less than the PTR method (Rs.3420.5/acre) to prepare land due to fewer tillage operations. DSR and PTR crop establishment costs (sowing and transplanting) vary widely across regions. DSR sowing costs Rs.1260/acre, about one-third of PTR transplanting (Rs.3787/acre). DSR has lower seed and seed treatment costs (43.5%) than PTR due to lower seed rates. DSR costs more for weeding and pest control than PTR. DSR weed and pest management costs are 6.4% and 19.2% higher than PTR. DSR plots require less family and hired labor (13.4 and 29.3 days/acre) than PTR plots (15.8 and 35.7 days/acre). DSR costs less to grow than PTR in UP (23.45%), MP (33.1%), AP (19.4%), and TS (38.7%). Savings are greatest in land preparation, sowing/transplanting, seed, and seed treatment.

Farmers prefer to prioritize rice yield when choosing a new technology or variety in the regions of average productivity. DSR plots yield 1777kg/acre less in MP than TPR plots (2043kg/acre). Lack of capacity building among farmers, including low educational attainment and limited access to extension services, may explain MP's lower yield under DSR adoption [35, 36]. UP, AP, and TS rice yields increased by 3.1%, 5.7%, and 6.4% in DSR compared to PTR. Many South Asian studies found that well-watered paddy crops in aerated soils yield more under DSR than PTR [26, 35, 37-39].

3.2. Impact of DSR-practice adoption

3.2.1. Quality of matching using a covariate balancing test

Before assessing the impact of DSR adoption, it is necessary to ensure that the distribution of pertinent variables is in equilibrium between those who adopted the DSR establishment method and those who have not. Before and after the matching, the comprehensive outcomes of the covariate balancing test for each state are presented in Table 3. In PSM, the standardized mean difference for all covariates is decreased by 32% to 46%, regardless of the matching algorithm employed. It signifies a significant decrease in overall bias achieved via matching. Furthermore, upon matching, the ρ-values of the likelihood ratio tests indicate that the joint significance of covariates is rejected, whereas it was not rejected before matching. Following matching, the pseudo-R^2 values for all three matching algorithms decrease substantially, irrespective of the states. The low pseudo-R^2, insignificant ρ-values of the likelihood ratio test, and low mean standardized bias suggest that the propensity score effectively achieves a state of equilibrium in the distribution of covariates between the two groups (DSR adopters and non-adopters).

Table 3. Covariate balancing test before and after matching.

		Matching algorithm	Pseudo-R2 before matching	Pseudo-R2 after matching	$\rho > \chi 2$ before matching	$\rho > \chi 2$ after matching	Mean standardized bias before matching	Mean standardized bias after matching	(Total)% |bias| reduction
**Adoption to DSR**	**Uttar Pradesh**	NNM	0.177	0.048	0.017	0.267	33.19	20.52	38.16
		RM	0.165	0.044	0.024	0.283	32.56	22.09	32.15
		KBM	0.183	0.039	0.032	0.304	35.65	20.98	41.16
	**Madhya Pradesh**	NNM	0.154	0.052	0.025	0.299	28.89	18.17	37.1
		RM	0.167	0.046	0.033	0.276	29.92	18.99	36.54
		KBM	0.172	0.056	0.028	0.256	27.17	17.50	35.59
	**Andhra Pradesh**	NNM	0.155	0.036	0.022	0.299	35.26	21.71	38.44
		RM	0.149	0.032	0.037	0.354	37.11	21.72	41.46
		KBM	0.143	0.033	0.031	0.324	35.71	20.04	43.89
	**Telangana**	NNM	0.168	0.045	0.029	0.273	33.66	18.27	45.72
		RM	0.162	0.041	0.03	0.318	30.45	17.16	43.65
		KBM	0.154	0.036	0.034	0.307	29.18	17.27	40.83

3.2.2. *Impact assessment*

The average impact of DSR adoption in different geographies (UP, MP, AP, and TS) is estimated using the PSM method. The study uses three matching algorithms, i.e., nearest neighbor matching (NNM), kernel-based matching (KBM), and radius matching (RM), to measure the average treatment effect on treated (ATT). ATT measures the difference in outcomes between DSR adopters and non-adopters. Table S1-S4 represents the impact of DSR adoption on the cost of cultivation, yield, and income for UP, MP, AP, and TS, respectively. The results support our findings and inferences in descriptive analysis.

Table 4 shows the comparative impact of DSR adoption in 4 different geographies. The results indicate that land preparation savings are highest in TS (Rs3480/acre) and lowest in AP (Rs1586/acre). In TS, farmers perform intensive land preparation activities like tilling (Primary and secondary tillage), puddling, harrowing, and leveling to maintain a water level of 3-5cm for up to 90 days in PTR. However, the DSR method does not require extensive land preparation activities. Hence, investment is less in DSR across all locations than in PTR. The seed and seed treatment cost is also saved (Rs279/acre to Rs545/acre based on the location) in DSR. This trend is found in all four geographies. In PTR, the seed rate (25-30 kg/acre) is 2.5 to 3 times higher than DSR (8-10 kg/acre). Hence, farmers need to invest more in seed costs under PTR than DSR.

Table 4. Comparative analysis of PSM outcomes

**Variables**	**UP**	**MP**	**AP**	**TS**
	**ATT**			
Land preparation cost (Rs/acre)	-2908***	-1769***	-1586***	-3480***
	(47.84)	(49.12)	(60.56)	(128.08)
Seed and seed treatment cost (Rs/acre)	-279**	-545**	-533**	-516**
	(71.01)	(75.89)	(60.60)	(56.49)
Crop establishment cost (Rs/acre)	-1580***	-1705***	-2120***	-1951***
	(222.34)	(244.83)	(266.24)	(140.18)
Total fertilizer cost (Rs/acre)	-466	-772**	-335	-529
	(83.49)	(110.79)	(51.20)	(67.28)
Irrigation cost (Rs/acre)	-629**	-728**	-1092***	-1203***
	(74.22)	(141.88)	(209.49)	(125.81)
Weed control cost (Rs/acre)	161**	307**	241**	322**
	(36.92)	(66.45)	(44.34)	(60.89)
Pest control cost	398**	37.33*	294**	207**
	(61.27)	(17.47)	(69.39)	(67.96)
Harvesting cost	-58.67	-37.67	-115.00	-317.67
	(56.09)	(23.51)	(78.90)	(42.84)
Post-harvest cost	-104.00	-345.00	-308.33	-722.00
	(78.80)	(166.04)	(87.87)	(156.87)
Total labor (days/acre)	-8.02***	-6.92***	-7.64***	-7.70***

	(1.04)	(1.10)	(1.19)	(0.40)
Family labor (days/acre)	-3.18**	-3.15**	-1.69**	-2.69**
	(0.34)	(0.48)	(0.39)	(0.28)
Total cost (Rs/acre)	-6867***	-5552***	-5043***	-7717***
	(679.67)	(447.44)	(500.08)	(436.37)
Rice yield (kg/acre)	58	-178**	92.33**	125.67**
	(48.28)	(78.06)	(30.59)	(36.35)
Income from rice (Rs/acre)	6238***	5693***	5009***	8134***
	(578.33)	(388.71)	(424.23)	(1293.67)

*=significant at 10%; **=significant at 5%; ***=significant at 1%

However, in DSR, farmers tend to protect the seeds from insect and pest attacks for better germination, and this is why pre-treatment of the seeds with insecticide and pesticide increases the seed treatment cost in DSR. Farmers save the crop establishment cost of Rs1580/acre to Rs3172/acre based on the location by adopting DSR. We observed that the saving is maximum in MP and lowest in UP. In PTR, growing the nursery on a piece of land, transporting the nursery to different plots, and transplanting the seedlings are labor and input (fertilizer and irrigation) intensive processes, which increases the cost of cultivation in PTR than DSR. Therefore, the additional savings in crop establishment are higher in TS and AP than in UP and MP. In all the locations except MP, fertilizer costs play an insignificant role in the PTR and DSR methods. In MP, adopters of DSR experienced less fertilizer cost of Rs772/acre due to improved nutrient efficiency with split application than DSR non-adopters.

Irrigation costs across all locations are significantly lower in DSR than in PTR despite more irrigation per season. In PTR, farmers maintain standing water of 3-5cm till the grain filling stage (75-90 days based on variety), while in DSR, farmers provide intermittent water (Alternate wetting and drying) based on the soil moisture condition. Hence, the overall water requirement in DSR is 30-50% less than PTR, resulting in less irrigation cost in DSR than PTR. In MP, due to heavy soil conditions, the water-holding capacity is higher. Similarly, clay loam soil in UP also retains more moisture. Hence, the water requirement or frequency of irrigation to maintain 3-5cm standing water is less. However, in AP and TS, the soil is sandy/silty loam

in nature. Therefore, farmers need to provide frequent irrigation to maintain water logging conditions in PTR fields. Hence, DSR adoption in AP and TS is more profitable regarding irrigation cost than PTR.

Unlike irrigation, the weed and pest control costs are higher in DSR. Due to a lack of intensive tillage activities and alternate wetting-drying irrigation practices, the weed pressure remains high in DSR. Hence, pre and post-emergence weedicides are applied in DSR plots to rescue the seedlings from weed pressure and nutrient competition. Besides weed, pest attacks are also more prevalent in DSR than in PTR. Therefore, the cost associated with plant protection is higher in DSR than in PTR. Results also indicate that farmers with DSR adoption can save 9.33 to 11.20 man-days (Hired and Family labor) compared to DSR farmers.

Minimum land preparation and absence of transplanting activity in DSR save men and women days. Our results show that the total cost of cultivation is significantly less (Rs5043/acre to Rs7718/acre) in DSR than in conventional PTR farming. Less cost associated with land preparation, seed and seed treatment, crop establishment, and irrigation results in reduced cost of cultivation under DSR practice. Except for MP, an increased yield of 58kg/acre, 92kg/acre, and 126kg/acre under DSR is reported in UP, AP, and TS, respectively. However, states in peninsular regions (AP and TS) achieve a significantly higher yield in DSR than PTR, which might result from the nature of soils having a good balance of sand and silt with clay. The clay component helps retain nutrients, while the sand component of the soil maintains good aeration of roots. At the same time, in MP, the yield under DSR is significantly lower (179kg/acre) than PTR, which might be because of heavy clayey soil conditions and related complications like delayed emergence, increased risk of seedling diseases, conducive environment for weed growth, compaction and poor drainage. Nevertheless, results indicate that the net income from paddy cultivation using the DSR method is significantly higher, irrespective of different geographies. For states like UP, MP, and AP, the net income from DSR adoption varies

between Rs5009/acre to Rs6237/acre, while in TS, the net income from DSR adoption is Rs 8133/acre higher than PTR. The relatively higher return on investment in TS can be ascribed to larger land holdings and more mechanization, significantly reducing costly labor while a better market channel fetches good prices for paddy. With increased yield and better price realization due to early harvest, DSR is more profitable in TS than in other geographies.

3.2.3. ESR-based paddy yield impact

The findings identified that the PSM technique may be biased due to unobservable variables. Consequently, this investigation has implemented the FILM-based ESR model to mitigate the inherent biases and reliability issues linked to the PSM model. Table 5 illustrates the average treatment effect of DSR adoption on paddy yield in both actual and counterfactual scenarios. The data is derived from the ESR method. Compared to non-DSR adopters, DSR adopters attain increased yields in UP, AP, and TS. However, conventional PTR farmers achieve better MP yields (10.69%) than DSR farmers. In UP, AP, and TS, if DSR non-adopters decide to adopt DSR, their yield can improve by 3.58%, 3.15%, and 4.49%, respectively.

Table 5. Average treatment effects of DSR on rice yield, cost of cultivation, and net income in four different states in India

Variables	Type of farmers and treatment effect	Uttar Pradesh			Madhya Pradesh			Andhra Pradesh			Telangana		
		Decision stage		ATEs	Decision stage		ATEs	Decision stage		ATEs	Decision stage		ATEs
		To adopt DSR farming	Not to adopt DSR farming		To adopt DSR farming	Not to adopt DSR farming		To adopt DSR farming	Not to adopt DSR farming		To adopt DSR farming	Not to adopt DSR farming	
Yield (kg/acre)	DSR adopting farmers	1775	1718	57	1806	1986	-180**	2671	2583	88*	2563	2457	106*
	DSR non-adopting farmers	1764	1703	61	1823	1999	-176**	2654	2573	81**	2555	2445	110*
	Heterogeneous effect	11	15	-4	-17	-13	-4	17	10	7	8	12	-4
Cost of cultivation	DSR adopting farmers	18251	25339	-7088***	17513	23105	-5592***	22347	27104	-4757***	17422	24932	-7510***
	DSR non-adopting farmers	18443	25603	-7160***	17654	23448	-5794***	22587	27325	-4738***	17804	25265	-7461***
	Heterogeneous effect	-192	-264	72	-141	-343	202	-240	-221	-19	-382	-333	-49
Net income	DSR adopting farmers	19706	14036	5670***	22521	17103	5418***	39115	34753	4362***	42634	34659	7975***
	DSR non-adopting farmers	19432	13803	5629***	21987	16653	5334***	38606	34345	4261***	42217	34387	7830***
	Heterogeneous effect	274	233	41	534	450	84	509	408	101	417	272	145

*=significant at 10%; **=significant at 5%; ***=significant at 1%

Table 6 provides parameter estimates for the yield effects of the DSR adoption on a double-log specification of the Production Function. The Cobb-Douglas specification could not be rejected, as indicated by the Wald test in the lower panel of Table 6. The estimated covariance terms are also displayed in the bottom portion of Table 6. Statistical analysis indicates heterogeneity, which could result in biased estimates if not corrected. The IMR coefficient is negative, indicating that the estimates would have been downward biased if the correction had not been made. In addition to the inputs used in rice cultivation, the analysis considers variations in several socio-economic and demographic explanatory variables (e.g., age,

farm experience, assured irrigation facility, education of the decision maker, etc.) in our criterion (selection) model, to minimize the biases in outcomes. The criterion function (selection equation in the model) and the regime equations are concurrently estimated in the ESR method to determine whether an observation belongs to a particular regime. The criterion equation includes all explanatory and instrumental variables [32]. In this study, we employed 11 socio-economic and demographic variables (Table 6) as instruments in the criterion function.

Table 6. Endogenous switching regression outcomes for paddy yield across four paddy growing states in India.

**Variables**	**Uttar Pradesh**			**Madhya Pradesh**			**Andhra Pradesh**			**Telangana**		
	**Criterion function**	**Regime equation**		**Criterion function**	**Regime equation**		**Criterion function**	**Regime equation**		**Criterion function**	**Regime equation**	
		**DSR**	**Conventional PTR**		**DSR**	**Conventional PTR**		**DSR**	**Conventional PTR**		**DSR**	**Conventional PTR**
Land preparation cost (Rs/acre), log	-0.662**	0.068	0.127**	-0.615**	0.073	0.143**	-0.585**	0.071*	0.167**	-0.666	0.176*	0.112**
	(0.218)	(0.052)	(0.043)	(0.226)	(0.056)	(0.041)	(0.342)	(0.021)	(0.055)	(0.378)	(0.066)	(0.054)
Seed and seed treatment cost (Rs/acre), log	-0.411**	-0.072**	0.056	-0.781	-0.092**	0.078	-0.693	-0.112**	0.061	-0.558	-0.121**	0.045**
	(0.156)	(0.019)	(0.018)	(0.245)	(0.081)	(0.033)	(0.331)	(0.039)	(0.031)	(0.121)	(0.049)	(0.021)
Crop establishment cost (Rs/acre), log	-0.217	0.031	0.043	-0.229	0.027	0.042	-0.231	0.022	0.031	-0.245	0.021	0.033
	(0.167)	(0.023)	(0.032)	(0.154)	(0.022)	(0.038)	(0.176)	(0.019)	(0.026)	(0.089)	(0.016)	(0.024)
Total fertilizer cost (Rs/acre), log	-0.539**	0.144**	0.181**	-0.482	0.212**	0.241**	-0.462	0.351**	0.391**	-0.473	0.366**	0.411**
	(0.222)	(0.041)	(0.054)	(0.255)	(0.063)	(0.079)	(0.243)	(0.101)	(0.107)	(0.253)	(0.118)	(0.128)
Irrigation cost (Rs/acre), log	-0.714**	0.187***	0.265***	-0.726	0.172***	0.247***	-0.679	0.234***	0.337***	-0.688	0.229***	0.328***
	0.298	0.064	0.089		0.044	0.088		0.073	0.099		0.097	0.134
Weed control cost (Rs/acre), log	-0.629***	0.272***	0.116**	-0.635	0.287***	0.104**	-0.661	0.283***	0.114***	-0.614	0.301***	0.109**

	(0.331)	(0.079)	(0.043)	(0.335)	(0.078)	(0.041)	(0.399)	(0.087)	(0.037)	(0.415)	(0.124)	(0.054)
Pest control cost (Rs/acre), log	-0.547*	0.087	0.093*	-0.543	0.107	0.114*	-0.528	0.076	0.084*	-0.544	0.118	0.126*
	(0.246)	(0.058)	(0.039)	(0.262)	(0.071)	(0.052)	(0.272)	(0.069)	(0.044)	(0.321)	(0.069)	(0.067)
Total labor cost (Rs/acre), log	-0.436**	0.013	0.227**	-0.449	0.011	0.224**	-0.417	0.012	0.218**	-0.439	0.014	0.222**
	(0.183)	(0.011)	(0.079)	(0.191)	(0.008)	(0.086)	(0.233)	(0.007)	(0.089)	(0.241)	(0.011)	(0.032)
Age of household head	0.028			0.032			0.022			0.025		
	(0.024)			(0.026)			(0.021)			(0.021)		
Education level (Years)	0.016**			0.018**			0.025**			0.029**		
	(0.004)			(0.007)			(0.008)			(0.011)		
Household size	0.015			0.009			-0.014**			0.007		
	(0.011)			(0.006)			(0.006)			(0.006)		
Community organization(s) member (%)	0.033**			0.027**			0.031**			0.033**		
	(0.012)			(0.013)			(0.011)			(0.011)		
Farm experience	0.013			0.015			0.015			0.015*		
	(0.008)			(0.011)			(0.009)			(0.007)		
Assured irrigation	-0.026**			-0.022**			-0.018**			-0.037**		
	(0.011)			(0.009)			(0.006)			(0.009)		
Livestock ownership (%)	0.018			0.015			0.013			0.015		
	(0.013)			(0.011)			(0.011)			(0.011)		
Smartphone ownership	0.024			0.027			0.024			0.027		
	(0.02)			(0.022)			(0.020)			(0.022)		
Extension service	0.037**			0.017*			0.025*			0.028*		
	(0.012)			(0.006)			(0.008)			(0.007)		
Crop insurance	0.019**			0.008			0.014**			0.018		
	(0.004)			(0.004)			(0.004)			(0.014)		

Total landholding (acre)	0.017			0.028**			0.016			0.016**		
	(0.013)			(0.007)			(0.014)			(0.007)		
Constant	-6.116	4.165	5.769	-7.265	4.339	5.934	-8.413	5.091	6.004	-7.534	5.567	4.659
	1.634											
LR (Wald test) for independent equations	187.18 (p-value less than 0.001)											

*=significant at 10%; **=significant at 5%; ***=significant at 1%

The parameter estimates of the criterion function are presented in Table 6. Costs associated with land preparation, seed and seed treatment, crop establishment, irrigation, weed control, fertilizer, and labor costs are the significant factors influencing the adoption of the DSR method across different geographies (UP, MP, AP, and TS). Furthermore, the adoption of DSR is significantly influenced by factors such as the farmer's education, community organization membership, assured irrigation, and extension service.

Farmers with more formal education are more likely to understand the information provided by agricultural experts and extension agents, enabling them to make well-informed judgments on new technology adoption. In addition, farmers with a formal education are more adept at forecasting the possible consequences of future technologies on agricultural productivity and financial viability. Therefore, there is a positive association between the educational level of the household head and their willingness to adopt innovative and sustainable farming practices, such as DSR. This observation is consistent with the findings published by Duraisamy [40], Idrisa et al. [41], and Huffman [42]. Adeoti [43] and Nonvide [44] found that farmers with greater levels of education demonstrate improved capacities to adopt and efficiently utilize sustainable technology.

Membership in a group or organization is a reliable measure of social capital. Social networks facilitate the exchange of knowledge and promote peer-to-peer learning among farmers. Social organizations functioned as an informal means of providing insurance during periods of distress. When group members endorse deploying new technology, it creates a favorable mindset among farmers, facilitating technology acceptance. This could explain why farmers with a group membership are more likely to adopt DSR practices.

Assured irrigation negatively affects the adoption of DSR technology. It indicates that farmers with assured access to irrigation choose PTR. The DSR method is primarily designed to conserve water in rice cultivation, minimize methane emissions from paddy fields, and improve soil health. Nevertheless, farmers who have assured irrigation are mostly unaware of the detrimental impacts of flood irrigation on soil and the ecosystem [43, 45-47]. Furthermore, in some states, the state government offers electricity for irrigation at a reduced cost or for free, which motivates farmers to continue flood irrigation based on the PTR method rather than adopt the water-saving DSR method.

Farmers acquire knowledge about the latest technologies by engaging with the extension agent. Through direct connection with extension personnel, farmers can resolve any uncertainties they may have regarding the technology. The reliable guidance provided by extension agents on achieving higher yields while minimizing cultivation expenses strongly motivates farmers to adopt DSR in rice farming.

The findings also suggest that an increase in the use of crop insurance has a favorable effect on the adoption of DSR for the farmers in UP and AP. Farmers who adopt DSR instead of conventional PTR also believe that if their crop yield is negatively affected by their lack of proficiency in adopting the new technique, crop insurance will offer an extra layer of protection against the resulting losses. Utilizing a certain risk management instrument encourages the adoption of other risk management solutions.

The results in Table 6 also suggest that landholding size positively impacts the adoption of DSR in MP and TS. This discovery is consistent with the results of prior investigations [48-52]. The farmers in UP and AP are mostly smallholders. They believe that they cannot attain food security and expected income if they lose their average yield due to adopting new technology. Conversely, large landowners in MP and TS allocate their land under both interventions (PTR and DSR). They are confident that they can recoup their income and guarantee food security through PTR despite the potential decrease in yield as a result of the implementation of DSR, given their relatively large farm size. The coefficients in the DSR and PTR methods exhibit some significant differences when considering various factors. In the DSR method, the weed control cost has the highest positive elasticity. A 10% increase in weed control cost enhances paddy yield by 2.72%, 2.82%, 2.83%, and 3.01% in UP, MP, AP, and TS, respectively. The weed issue appears to be more prevalent in DSR [25]. Controlling weeds, particularly at the seedling stage, is crucial to achieving good yield through DSR practice. Hence, pre and post-emergence herbicide application and maintaining moist conditions till 30 days after sowing is recommended to get optimum yield in the DSR method. Our discovery aligns with the findings of Antralina et al. [53], who discovered that rice yields are diminished by vegetation competition. In order to enhance rice yields, the authors recommend that farmers employ either chemicals or manual labor to manage plants. The cost of weed control also has a positive, albeit minor, impact on rice production under PTR. A 1% increase in weed control costs results in a 0.12%, 0.10%, 0.11%, and 0.11% increase in paddy yield in UP, MP, AP, and TS. Like weed, irrigation cost also shows a positive elasticity with paddy productivity across all states. Giving frequent irrigation in DSR plots also increases the paddy yield. Frequent irrigation rescues the crops from stress and controls the weeds. Soil water holding capacity is also crucial in irrigation amount and schedule. UP and MP have sandy loam and black soil with better water-holding capacity. Hence, these areas require less irrigation than AP and TS, which

have red lateritic soil with less water-holding capacity. Hence, compared to UP and MP, an increase in irrigation cost enhances the chance of paddy yield in AP and TS. Similarly, the fertilizer cost shows positive elasticity with yield across all the locations. A 10% increase in fertilizer cost enhances the paddy yield by 1.44%, 2.12%, 3.51%, and 3.66%. UP and MP contain more fertile soil compared to AP and TS. Hence, an increase in fertilizer rate, particularly the N-fertilizer, enhances the paddy yield significantly. UP contains alluvial soil, which is more fertile than others. Still, the N-content in the soil is low. Hence, the application of N-fertilizer increases the paddy yield. Like DSR, increased irrigation, fertilizer, and weed control costs augment the paddy yield in PTR across all geographies. In AP and TS, the land preparation costs in DSR also show positive elasticity with paddy yield: a 1% increase in land preparation cost augments the productivity of paddy by 0.07% and 0.18% in AP and TS. Due to lower water holding capacity and undulating land in AP and TS than in UP and MP, farmers form permanent bunds, do land leveling, and sometimes perform mild puddling activity to improve the soil water holding capacity. The DSR method exhibits positive elasticity for total labor: a 1% increase in total labor cost results in a rise in paddy yield of 0.22%-0.23% across all geographies. In DSR, labor cost shows insignificant elasticity with paddy productivity, which signifies that labor productivity increases with DSR adoption. The yield of rice is adversely affected by the cost of seed and seed treatment using the DSR method. Seed and seed treatment costs across all geographies significantly reduce paddy production under DSR. Kumar & Ladha [25] and Yadav et al. [54] have identified that an optimal seed rate is required for optimal paddy production in DSR. The yield can decrease if the seed quantity is above the optimal level. The higher number of seedlings/plants in a unit area, intense competition with plants and minimizes the yield.

4. Conclusion

The rice transplantation into puddled soil resulted in decreased soil health (creating a hard pan at shallow depth) and the underground water table. DSR presents itself as a feasible solution that addresses the issues of labor and water scarcity while also reducing greenhouse gas emissions. The DSR method could be a feasible substitute for the PTR method. Indian farmers are transitioning from the PTR approach to the DSR method. DSR can serve as a compelling substitute for PTR to decrease the labor requirement and lower cultivation expenses. This study has examined the impact of DSR adoption among farmers from four rice-producing states in India using the PSM approach. The effects of selection bias and heterogeneity caused by the DSR method are measured using the ESR method. The novel contributions of this work include the measure of DSR's economic suitability in four agro-climatic geographies. Also, the ESR methodology enables a more comprehensive understanding of the specific advantages of a farmer transitioning from the PTR to DSR in paddy production.

Both the PSM and ESR-based findings suggest that adopting DSR can lead to variations in paddy yield depending on agro-climatic conditions. Specifically, the yield increased in UP, AP, and TS, while it decreased in MP. However, the net income from paddy farming experienced a significant increase irrespective of state/agro-climatic conditions. The results also suggest that increasing investments in weed management, irrigation, and fertilizer can lead to higher yields in DSR. Among the socioeconomic variables, education, community organization membership, extension services, and crop insurance positively impact DSR adoption, while assured irrigation affects DSR adoption negatively.

India is currently facing substantial deficiencies in water and manpower, which are crucial resources for agricultural development, particularly in paddy production. For decades, farmers in India have employed the PTR establishment method to cultivate rice. Nevertheless, as the

water table steadily decreases in numerous regions of India, it is imperative to advocate for adopting resource-conserving technologies like DSR to guarantee food security for present and future generations. The findings of this study suggest that policymakers should create incentives to promote the adoption of DSR. The DSR approach can enhance the environment by reducing tillage or soil turnover, preventing soil erosion, and minimizing water usage. Policies designed to enhance DSR should prioritize the development of operator skills in weed management through the instruction of optimal management techniques. Our study also demonstrates the necessity for implementing policies and measures to improve the acceptance and utilization of DSR among individuals who have not yet adopted it.

The study has certain limitations. We could not account for variations in the quality of seed, labor, and land, which could have influenced the differences in yields due to limitations in the available data. Future research could focus on linkages between rural economies, extension approaches, and social networks. Additionally, it would be beneficial for future research to examine the environmental consequences, soil properties, and productivity improvements that result from DSR, in addition to the analysis of yield and cost implications. These factors have wide-ranging benefits for society.

Acknowledgments

We acknowledge the support of our field team for collecting primary data to support this study.

References

1. GRiSP (Global Rice Science Partnership). Rice Almanac: Source Book for One of the Most Important Economic Activities on Earth, 4th ed. (2013) http://ageconsearch.umn. edu//handle/164484
2. FAO. FAOSTAT Database. Food and Agriculture Organization of the United Nations (FAO), Rome, Italy (Retrieved 10 May 2020), (2018) http://www.fao.org/faostat/en/.

3. Valera H. & Bali´e J. The Outlook of the Rice Economy. International Rice Research Institute (IRRI), Los Banos, Philippines, Forthcoming (2020)
4. Bouman, BAM. & Tuong TP. Field water management to save water and increase its productivity in irrigated rice. *Agricultural Water Management*. 49, pp. 11–30 (2001) https://doi.org/ 10.1016/S0378-3774(00)00128-1
5. Farooq M., Siddique KH., Rehman H., Aziz T., Lee DJ. & Wahid A. Rice direct seeding: experiences, challenges and opportunities. *Soil and Tillage Research*. 111(2), pp. 87–98 (2011) https://doi.org/10.1016/j.still.2010.10.008.
6. Mahajan G., Kumar V. & Chauhan BS. Chapter 3 Rice production in India. (Springer, 2017)
7. Singh A. K, Choudury B. U. & Bouman B. A. M. Chapter 3 The effect of rice establishment techniques and water management on crop-water relations “Water-Wise Rice Production”. (IRRI, 2002)
8. Singh Y., Singh G., Johnson D. & Mortimer M. Chapter 6 Changing from transplanted rice to direct seeding in the rice-wheat cropping system in India. (IRRI, 2005)
9. Bhatt R. & Kukal SS. Resource conservation techniques for mitigating the water scarcity problem in the Punjab, India – a review. *International Journal of Earth Sciences and Engineering*. 7(1), pp. 309-316 (2014)
10. Dingkuhn M, de Vries Penning FWT, De Datta SK. & van Laar HH. Chapter 5 Concepts for a new plant type for direct seeded flooded tropical rice. (IRRI, 1991).
11. Sarkar RK. & Das S. Yield of rainfed lowland rice with medium water depth under anaerobic direct seeding and transplanting. *Tropical Science*. 43, pp. 192–198 (2003). 10.1002/ts.117

12. Islam S., Gathala MK., Tiwari TP., Timsina J., Laing AM., Maharjan, S., et al. Conservation agriculture based sustainable intensification: increasing yields and water productivity for smallholders of the Eastern Gangetic Plains. *Field Crops Research*. 238, pp. 1-17 (2019) https://doi.org/10.1016/j.fcr.2019.04.005
13. Ishfaq M., Farooq M., Zulfiqar U., Hussain S., Akbar N., Nawaz A. & Anjum SA. Alternate wetting and drying: a water-saving and ecofriendly rice production system. *Agricultural Water Management*. 241, pp. 106363 (2020) https://doi.org/10.1016/j.agwat.2020.106363.
14. Chakraborty D., Ladha, JK., Rana DS., Jat ML., Gathala MK., Yadav S., et al. A global analysis of alternative tillage and crop establishment practices for economically and environmentally efficient rice production. *Scientific Reports*. 7(1), pp. 1-11(2017) https://doi.org/10.1038/s41598-017-09742-9.
15. Carrijo DR., Lundy ME. & Linquist BA. Rice yields and water use under alternate wetting and drying irrigation: a meta-analysis. *Field Crops Research*. 203, pp. 173–180 (2017). https://doi.org/10.1016/j.fcr.2016.12.002.
16. Kar I., Mishra A., Behera B., Khanda C., Kumar V. & Kumar A. Productivity trade-off with different water regimes and genotypes of rice under non-puddled conditions in Eastern India. *Field Crops Research*. 222, pp. 218-229 (2018). https://doi.org/10.1016/j.fcr.2017.10.007.
17. Gopal R., Jat RK., Malik RK., Kumar V., Alam MM., Jat ML., et al. Direct Dry Seeded Rice Production Technology and Weed Management in Rice Based Systems. (Technical Bulletin. International Maize and Wheat Improvement Center, 2010).
18. Gupta RK., Ladha JK., Singh S., Singh R., Jat ML., Saharawat Y., et al. Series 8 Production technology for direct seeded rice. (Technical Bulletin of Rice–Wheat Consortium for the Indo-Gangetic Plains, 2006).

19. Ladha JK., Kumar V., Alam MM., Sharma S., Gathala M., Chandna P., et al. Chapter 1 Integrating crop and resource management technologies for enhanced productivity, profitability, and sustainability of the rice-wheat system in South Asia. (IRRI, 2009)
20. Xu L., Yuan S., Wang X., Yang G., Xiangcheng P. & Yu X., et al. Productivity and global warming potential of direct seeding and transplanting in double-season rice of central China. *Food and Energy Security*. 12, pp. e419 (2023). https://doi.org/10.1002/fes3.419
21. Qian H., Zhu X., Huang S., Linquist B., Kuzyakov Y., Wassmann R., et al. Greenhouse gas emissions and mitigation in rice agriculture. *Nature Reviews Earth & Environment*. 4, pp. 716–732 (2023). https://doi.org/10.1038/s43017-023-00482-1
22. Pathak H., Sankhyan S., Dubey DS., Bhatia A. & Jain N. Dry direct-seeding of rice for mitigating greenhouse gas emission: field experimentation and simulation. *Paddy and Water Environment*. 11, pp. 593-601 (2013). https://doi.org/10.1007/s10333-012-0352-0
23. Johnkutty I, Mathew G. & Mathew J. Comparison between transplanting and direct-seeding methods for crop establishment in rice. *Journal of Tropical Agriculture*. 40, pp. 65–66 (2002)
24. Can ND. & Xuan VT. Chapter 2 Environmental conditions as determinants of direct seeding techniques in different ecosystems in the Mekong Delta of Vietnam. Proceedings of the International Workshop on Direct Seeding in Asian Rice Systems: Strategic Research Issues and Opportunities, 2002)
25. Kumar V. & Ladha JK. Direct seeding of rice: recent developments and future research needs. *Advances in Agronomy*. 111, pp. 297–413 (2011) https://doi.org/10.1016/B978-0-12-387689-8.00001-1

26. Yadav S., Gill MS., Humphreys E., Kukal SS. & Walia US. Effect of water management on dry seeded and puddled transplanted rice. Part 1: Crop performance. *Field Crops Research.* 120(1), pp. 112–122 (2011). https://doi.org/10.1016/j.fcr.2010.09.002
27. Wollni M. & Brümmer B. Productive efficiency of specialty and conventional coffee farmers in Costa Rica: Accounting for technological heterogeneity and self-selection. *Food Policy*. 37, pp. 67-76 (2012) https://doi.org/10.1016/j.foodpol.2011.11.004
28. Dehejia RH. & Wahba S. Propensity score-matching methods for non-experimental causal studies, *The Review of Economics and Statistics*. 84, pp. 151–161 (2002) https://doi.org/10.1162/003465302317331982.
29. Rosenbaum PR. & Rubin DB. Constructing a control group using multivariate matched sampling methods that incorporate the propensity score, *The American Statistician.* 39, 33–38 (1985) https://doi.org/10.1080/00031305.1985.10479383.
30. Greene W. Econometric Analysis. Prentice Hall, Englewood Cliffs. (2012)
31. Fuglie KO. & Bosch DJ. Economic and environmental implications of soil nitrogen testing: a switching-regression analysis. American Journal of Agricultural Economics. 77, pp. 891–900 (1995) https://doi.org/10.2307/1243812
32. Lokshin M. & Sajaia Z. Maximum likelihood estimation of endogenous switching regression models. *Stata Journal*. 4, pp. 282–289 (2004).
33. Noltze M., Schwarze S. & Qaim M. Impacts of natural resource management technologies on agricultural yield and household income: the system of rice intensification in Timor Leste. *Ecological Economics*. 85, pp. 59–68 (2013).
34. Alene A. & Manyong V. The effects of education on agricultural productivity under traditional and improved technology in northern Nigeria: an endogenous switching

regression analysis. *Empirical Economics*. 32(1), pp. 141–159 (2007) https://doi.org/10.1007/s00181-006-0076-3

35. Ali A., Erenstein O. & Rahut B. The impact of direct rice-sowing technology on rice producers' earnings: empirical evidence from Pakistan. *Development Studies Research*. 1(1), pp. 244–254 (2014) https://doi.org/10.1080/21665095.2014.943777
36. Singh S., Ladha JK., Gupta RK., Bhushan L. & Rao AN. (2008). Weed management in Aerobic rice systems under varying establishment methods. *Crop Protection*. 27(3), pp. 660-671. https://doi.org/10.1016/j.cropro.2007.09.012
37. Hobbs P., Singh Y., Giri G., Lauren J. & Duxbury J. Chapter 6 Direct-seeding and reduced tillage options in the rice-wheat system of the Indo-Gangetic Plains of South Asia. (IRRI, 2002).
38. Gill MS. Productivity of direct-seeded rice (Oryza sativa) under varying seed rates, weed control and irrigation levels. *The Indian Journal of Agricultural Sciences*. 78, pp. 766–770 (2008).
39. Singh U., Singh Y., Kumar V. & Ladha J. Evaluation and promotion of resource-conserving tillage and crop establishment techniques in the rice-wheat system of eastern India. (IRRI, 2009).
40. Duraisamy P. Changes in returns to education in India, 1983–94: by gender, age-cohort and location. *Economics of Education Review*. 21, pp. 609–622 (2002) https://doi.org/10.1016/S0272-7757(01)00047-4
41. Idrisa. Effects of adoption of improved maize seed on household food security in Gwoza Local government area of Borno state, Nigeria. *Global Journal of Science Frontier Research*

Agriculture & Biology. 12, pp. 7–12 (2012). https://journalofscience.org/index.php/GJSFR/article/view/508/2-Effects-of-Adoption-of-Improved-Maize-Seed_html. (Accessed 27 September 2023).

42. Huffman W. Chapter 7 Human Capital: education and agriculture. (Elsevier, 2001).
43. Adeoti IA. Factors influencing irrigation technology adoption and its impact on household poverty in Ghana. *Journal of Agriculture and Rural Development in the Tropics and Subtropics*. 109, 51–63 (2009).
44. Nonvide GMA., Sarpong TMG., Kwadzo H., Anim-Somuah F.& Gero FA. Farmers' perceptions of irrigation and constraints on rice production in Benin: a stakeholder-consultation approach. *International Journal of Water Resources Development*. 34, pp. 1001–1021 (2017). https://doi.org/10.1080/07900627.2017.1317631
45. Abdulai A., Owusu VC. & Bakang JEA. Adoption of safer irrigation technologies and cropping patterns: evidence from Southern Ghana. *Ecological Economics*. 70, 1415–1423 (2011). https://doi.org/10.1016/j.ecolecon.2011.03.004
46. Genius M., Koundouri P., Nauges C. & Tzouvelekas V. Information transmission in irrigation technology adoption and diffusion: social learning, extension services, and spatial effects. *American Journal of Agricultural Economics*. 96, 328–344 (2013). https://doi.org/10.1093/ajae/aat054
47. Koundouri P., Nauges C. & Tzouvelekas V. Technology adoption under production uncertainty: theory and application to irrigation technology. *American Journal of Agricultural Economics*. 88, 657–670 (2006). https://doi.org/10.1111/j.1467-8276.2006.00886.x
48. Tura M., Aredo D., Tsegaye W., Rovere R., Girma T., Mwangi W. & Mwabu G. Adoption and continued use of improved maize seeds: case study of central Ethiopia. *African Journal of Agricultural Research.* 5, pp. 2350–2358 (2010)

49. Houeninvo GH., Quenum CVC. & Nonvide GMA. Impact of improved maize variety adoption on smallholder farmers' welfare in Benin. *Economics of Innovation and New Technology*. 29, pp. 831–846 (2018) https://doi.org/10.1080/10438599.2019.1669331
50. Ali EB., Awuni JA. & Danso-Abbeam G. Determinants of fertilizer adoption among smallholder cocoa farmers in the Western Region of Ghana. *Cogent Food & Agriculture*. 4, pp. 1538589 (2018) https://doi.org/10.1080/23311932.2018.1538589
51. Abay KA., Berhane G., Taffesse AS., Abay K. & Koru B. Estimating input complementarities with unobserved heterogeneity: evidence from Ethiopia. *Journal of Agricultural Economics*. 69, pp. 495–517 (2018). https://doi.org/10.1111/1477-9552.12244
52. Mwangi M. & Kariuki S. Factors determining adoption of new agricultural technology by smallholder farmers in developing countries. *Journal of Economics and Sustainable Development*. 6, pp. 208–216 (2015).
53. Antralina M., Istina IN., Yuwariah Y. & Simarmata T. Effect of difference weed control methods to yield of lowland rice in the SOBARI. *Procedia Food Science*. 3, pp. 323–329 (2015) https://doi.org/10.1016/j.profoo.2015.01.035
54. Yadav S., Gill MS, & Kukal SS. Performance of direct-seeded basmati rice in loamy sand and in semi-arid sub-tropical India. *Soil and Tillage Research*. 97, pp. 229–238 (2007). https://doi.org/10.1016/j.still.2007.09.019
55. Sharma P., Tripathi RP. & Singh S. Tillage effects on soil physical properties and performance of rice–wheat-cropping system under shallow water table conditions of Tarai, Northern India. *European Journal of Agronomy*. 52, pp. 12–16 (2004) https://doi.org/10.1016/j.eja.2005.01.003

56. Sarkar RK. & Das S. Yield of rainfed lowland rice with medium water depth under anaerobic direct seeding and transplanting. *Tropical Science.* 43, pp. 192–198 (2003). https://doi.org/10.1002/ts.117

57. Rath BS., Misra RD., Pandey DS. & Singh VP. Effect of sowing methods on growth, productivity and nutrient uptake of wheat at varying level of puddling in rice. *Indian Journal of Agronomy*. 45, pp. 463–469 (2000).

58. Tripathi RP., Sharma P. & Singh S. Tillage index: an approach to optimize tillage in rice-wheat system. *Soil and Tillage Research*. 80, pp. 125–137 (2005) https://doi.org/10.1016/j.still.2004.03.004

59. Singh SK,, Bharadwaj V., Thakur TC., Pachauri SP., Singh PP. & Mishra AK. (2009) Influence of crop establishment methods on methane emission from rice fields. Current Science. 97, pp. 84–89 (2009).

Figure
Click here to access/download;Figure;Figure 1.docx

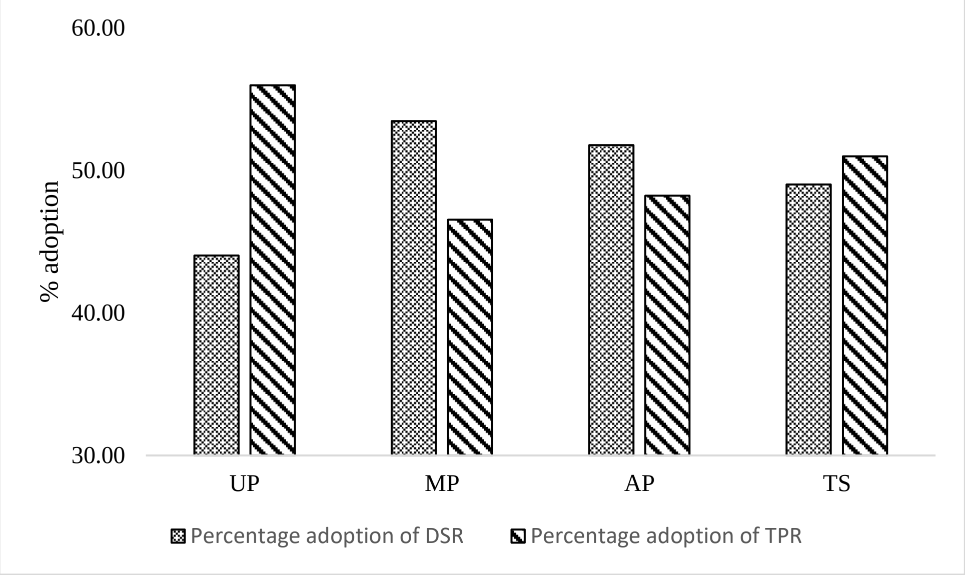

Figure 1. Percentage of surveyed farmers adopted DSR and PTR in different project locations.

Click here to access/download
Supporting Information
Supporting information.docx
