# Supplementary material for: Economic suitability of direct seeded rice across different geographies in India
Source: PLoS One. 2025 Apr 18;20(4):e0321472. doi: 10.1371/journal.pone.0321472 (PMC12007715; doi:10.1371/journal.pone.0321472)
Supplement: Attachment — Submitted filename: ATR.docx [file pone.0321472.s006.docx]

**Author’s Response to The Editor’s and Reviewer’s Comments**

Thank you very much for your time and efforts in reviewing the MS. We have addressed all the concerns. The point to point response to all of the comments of both reviewers are given below.

**Reviewer #1:**

**Comment**: The manuscript deals with direct seeded rice feasibility as a sustainable substitute for puddled transplanted rice in India’s paddy production. The findings are promising for farmers and policymakers aiming to address labor and water scarcity in agriculture while promoting eco-friendly practices. The researchers collected extensively data from 537 households cultivating rice through DSR and PTR. They followed PSM and ESR methodology for analysis for proper outcome of their study, however, I found some limitations in their study which needs to be improved before acceptance.

**Comment:**1. L47 and 48: Rewrite sentence

**Authors’ response:** Done, Thank You. We have rewritten the sentences. Please refer to line no.: 47 & 48.

**Comment:** 2.Table 2: Yield 9kg/acre) in case of UP is exactly same when farmers sown rice through DSR and PTR, what are the factors that contributed to equal yield (when sample size is quite good), while weed and pest problem occurrence were more in DSR? Please incorporate explanation in discussion section for better understanding of readers.

**Authors’ response:** **Done**, Thank You. In UP, the rice yield (kg/acre) in a recently adopted DSR (last 3 years) and PTR (Table 2) is tending to a significant difference. In the case of DSR, yield is 1793kg/acre while for PTR it is 1739kg/acre, indicating a better nutrient use efficiency in case of DSR. Furthermore, puddling in PTR can lead to soil compaction, which negatively affects root growth and water infiltration. On the other hand, DDSR's roots grow deeper and stronger as they are not confined to the puddled soil. This can lead to better nutrient uptake and overall plant health, as well as increased yield. Also, inherent wetting and drying cycles in absence of ponded water in DSR promotes better microbial growth in the soil. The symbiotic relationship between microbes and plant roots leads to better nutrient availability for the plant, leading to better plant growth and yield than PTR. We have included these in the discussions section. (Lines: 280-287).

Yes, we agree that the weed and pest manifestation is high in DSR. The problem worsens slowly with multiple cycles of DSR, where weed-seed-bank in deeper soil gets activated. However, these are less prominent in initial years and can be controlled by following a proper weed management schedule. Our study observed that farmers in UP are well-trained in managing weeds in DSR plots. They apply pre and post-emergence weedicides to manage the weed growth, which is prevalent in the first 30 days after sowing.

**Comment:**3. In discussion section, it is written that farmers of UP are mostly having small land holding, and still they got equal yield, yield is one of the major factors in choosing new technology, then why farmers are still hesitating in adopting new technology, any reasons, incorporate it?

**Authors’ response:** **Done**. We agree that the farmers of UP are mostly having small land holding and yield is one of the major factors in choosing new technology. However, we observed that the delay in monsoon has devastating impacts in sandy loam and sandy clay loam soils of UP. There is low seed germination and farmers need to invest more in seed procurement and require additional labor for gap-filling. Nevertheless, intense rainfall after few weeks of sowing damages the re-sown seed. Additionally, socio-psychological factors, like the risk of losing yield if they fail to control weeds, risk of social exclusion from the community if they adopt a new practice, and difficulty in machine harvesting as DSR plots mature 10-15 days earlier than PTR. These are among few important factors responsible for slow adoption of DSR over PTR in UP. We have mentioned these in the revised MS. Please refer to lines: 446-453.

**Comment:** 4. L468: The yield of rice is adversely affected by seed treatment; isn’t statement is contradictory?

**Authors’ response:** **Done.** Thank you for your suggestion. We have rewritten the sentence. Please refer to lines: 492-495.

**Comment:** 5: While the study mentions economic and agronomic outcomes, it does not extensively explore how socioeconomic factors, such as education, access to technology, and extension services, impact dDSR adoption and success?

**Authors’ response: Done.** The impact of socio-economic factors on DSR adoption over PTR has been extensively covered in our previous publication (Dey et al., 2023). Therefore, in this study, our focus was to identify the economic suitability of DSR across four geographies. However, we have identified the impact of socio-economic constraints on DSR adoption for all four geographies in this study as well. Please refer to lines: 389-396, 400-409, 412-416.

**Comment:**6. Compare data from farmers field and institute research trials located in these states across agro-climatic zones for better comparison, also targeted trials to identify specific adaptations needed for dDSR success? Tailoring techniques based on regional characteristics, such as soil type, climate, and water availability, could improve its suitability and encourage broader adoption.

**Authors’ response:** Thank you for your suggestions. In fact, we are planning in the similar line. We are also doing some experimental trials considering the differences in the soil type, water, and climatic characteristics in all four locations. Once we complete the 2-year rigorous data analysis from the experimental plots, we will draft a manuscript on the same line. Nevertheless, we have mentioned it as a limitation of current study. Please refer to lines: 535-537.

**Comment:**7. Consider additional data variables such as soil health metrics, irrigation water quality, farmer education levels, and labor skills. Including these factors in analyses would provide a more comprehensive understanding of the variables influencing dDSR success.

**Authors’ response:** Thank you for your suggestions. We need to perform soil and water analysis in all locations to include these parameters. In the future, we will work on this and develop a separate manuscript. However, we added this as a limitation of our study. Please refer to lines: 533-535.

**Reviewer #2**

**Comment:** The article needs through revision before considerations in the journal. I have gone through the ms and my commens is appended in the manuscript pdf. In my view, whole ms need to be re-write with suitable logic and data support. Most of places very old rreferences were given. In my view, delete all old references and cite relevant one. Avoild to use lumsum references and summary and conclusion should be presented in bullts form to better impact for international readers.

**Authors’ response:**	Thank you very much for your time and efforts in reviewing the MS. We have addressed all your concerns in revised MS Please, find point-to-point responses below and marked blue in revised MS draft.

**Comment: What happened about Punjab, Haryana and eastern states of India**

**Authors’ response:** Thank you for this suggestion. We concur that it would be advantageous to compare all rice-growing agro-climatic zones in a study. However, this study aimed to identify the economic suitability of DSR across four principal agro-climatic zones, each characterised by distinct soil types. These are our primary impact regions where we collaborate with farmers and various government departments. Moreover, the agricultural practices in these regions exhibit distinct variations. The government incentive for DSR cultivation in Punjab offers it a competitive advantage over traditional PTR methods. The land area in Punjab and Haryana is significantly larger than in these regions. In contrast, the meteorological and precipitation patterns in eastern India markedly diverge from those in these regions. Therefore, including Punjab, Haryana, and Eastern states in this analysis would not have yielded a comparable level playing field. Nonetheless, we acknowledge this as a weakness of the study and want to prepare a separate publication addressing it.

**Comment: Puddle transplanting rice (PTR)**

**Authors’ response:** Done. Thank You. Please refer to lines: 55-56.

**Comment: The**

**Authors’ response:** Done. Thank You. Please refer to line: 61.

**Comment: pl cite recent references instead of very old**

**Authors’ response:** Done. We have cited recent references in Table 1. (Line 73)

**Comment: Pl write in bulleted form for better readability to the international readers**

**Authors’ response:** Done, Thank you for your suggestion. We have added bulleted highlights as summary of the MS. Please find it in the Lines: 11-19.

**Comment: Use recent references and delete old an irrelevant one**

**Authors’ response:** Done. Recent references are included and the older ones have been deleted across the revised MS. For example, please refer to lines nos:575-588.
