# Supplementary material for: Economic suitability of direct seeded rice across different geographies in India
Source: PLoS One. 2025 Apr 18;20(4):e0321472. doi: 10.1371/journal.pone.0321472 (PMC12007715; doi:10.1371/journal.pone.0321472)
Supplement: Attachment — Submitted filename: Response to Reviewers.docx [file pone.0321472.s008.docx]

**Author’s Response to The Editor’s and Reviewer’s Comments**

Thank you very much for your time and efforts in reviewing the MS. We have addressed all the concerns. The point-to-point response to all of the comments of both reviewers is given below.

**Editor Comments are as follows:**

*Comment:* A rebuttal letter that responds to each point raised by the academic editor and reviewer(s). You should upload this letter as a separate file labeled 'Response to Reviewers'.

*Authors’ response:* Done. Thank you for the suggestion.

*Comment:* A marked-up copy of your manuscript that highlights changes made to the original version. You should upload this as a separate file labeled 'Revised Manuscript with Track Changes'.

*Authors’ response:* Done. Thank you for the suggestion.

*Comment:* An unmarked version of your revised paper without tracked changes. You should upload this as a separate file labeled 'Manuscript'.

*Authors’ response:* Done. Thank you for the suggestion.

**Journal Requirements:**

*Comment:* Please review your reference list to ensure that it is complete and correct. If you have cited papers that have been retracted, please include the rationale for doing so in the manuscript text, or remove these references and replace them with relevant current references. Any changes to the reference list should be mentioned in the rebuttal letter that accompanies your revised manuscript. If you need to cite a retracted article, indicate the article’s retracted status in the References list and also include a citation and full reference for the retraction notice.

*Authors’ response:* Done. We have not cited any paper that has retracted **Additional Editor Comments:**

*Comment:* Thank you for revise of manuscript and substantial improvement but still it has limitation in in discussion and conclusion part.

*Authors’ response:* Done. Thank you for your suggestion. We have rewritten the discussion and conclusion part. Please refer to lines no.: 220-473

*Comment:* Separate the result and discussion part in to different subheading so that result can be discussed well in in discussion part.

*Authors’ response:* Done. We have separated the results and discussion under different sub-headings. Please refer to line no.: 220-253, 267-297, 300-316, 322-450.

Conclusion is to be conclusive so it should be short with only research findings oriented and research way forwards.

*Authors’ response:* Done. We have rewritten the conclusion. Please refer to lines no.: 452-473.
